# Collagen type XIX regulates cardiac extracellular matrix structure and ventricular function

**DOI:** 10.1016/j.matbio.2022.03.007

**Published:** 2022-03-26

**Authors:** Ghazal Sadri, Annalara G. Fischer, Kenneth R. Brittian, Erin Elliott, Matthew A. Nystoriak, Shizuka Uchida, Marcin Wysoczynski, Andrew Leask, Steven P. Jones, Joseph B. Moore

**Affiliations:** a **Diabetes and Obesity Center,** University of Louisville School of Medicine, Louisville, KY, USA; b **Center for RNA Medicine,** Department of Clinical Medicine, Aalborg University, Copenhagen, Denmark; c **College of Dentistry,** University of Saskatchewan, Saskatoon, SK, Canada

**Keywords:** Collagen type XIX, Cardiac fibroblasts, Extracellular matrix structure, Cardiac function, Cardiomyocyte hypertrophy

## Abstract

The cardiac extracellular matrix plays essential roles in homeostasis and injury responses. Although the role of fibrillar collagens have been thoroughly documented, the functions of non-fibrillar collagen members remain underexplored. These include a distinct group of non-fibrillar collagens, termed, fibril-associated collagens with interrupted triple helices (FACITs). Recent reports of collagen type XIX (encoded by *Col19a1*) expression in adult heart and evidence of its enhanced expression in cardiac ischemia suggest important functions for this FACIT in cardiac ECM structure and function. Here, we examined the cellular source of collagen XIX in the adult murine heart and evaluated its involvement in ECM structure and ventricular function. Immunodetection of collagen XIX in fractionated cardiovascular cell lineages revealed fibroblasts and smooth muscle cells as the primary sources of collagen XIX in the heart. Based on echocardiographic and histologic analyses, *Col19a1* null (*Col19a1*^*N/N*^) mice exhibited reduced systolic function, thinning of left ventricular walls, and increased cardiomyocyte cross-sectional areas—without gross changes in myocardial collagen content or basement membrane morphology. *Col19a1*^*N/N*^ cardiac fibroblasts had augmented expression of several enzymes involved in the synthesis and stability of fibrillar collagens, including PLOD1 and LOX. Furthermore, second harmonic generation-imaged ECM derived from *Col19a1*^*N/N*^ cardiac fibroblasts, and transmission electron micrographs of decellularized hearts from *Col19a1*^*N/N*^ null animals, showed marked reductions in fibrillar collagen structural organization. *Col19a1*^*N/N*^ mice also displayed enhanced phosphorylation of focal adhesion kinase (FAK), signifying de-repression of the FAK pathway—a critical mediator of cardiomyocyte hypertrophy. Collectively, we show that collagen XIX, which had a heretofore unknown role in the mammalian heart, participates in the regulation of cardiac structure and function—potentially through modulation of ECM fibrillar collagen structural organization. Further, these data suggest that this FACIT may modify ECM superstructure via acting at the level of the fibroblast to regulate their expression of collagen synthetic and stabilization enzymes.

## Introduction

The myocardial extracellular matrix (ECM) provides structural context and facilitates force transmission. Yet, despite advancements in our understanding of the ECM in cardiovascular biology, the function of many matrix components remains largely unresolved. The earliest studies of cardiac ECM composition focused on the chief structural components of the interstitial matrix—namely, the fibrillar collagens [1]. In the heart, these consist predominantly of collagen types I and III, which, respectively, form dense, structural, rod-like fibers [2,3] and finer, elastic fiber networks [2,4,5]. Although these components of the cardiac ECM have well-characterized roles in cardiovascular health and disease [6], the importance of their lesser abundant associates, the non-fibrillar collagens, remains obscure.

Non-fibrillar collagens do not form fibrillar bundles but can associate with collagen types I and III fibrils to influence cardiac ECM organization [7,8]. Other studies implicate non-fibrillar collagens and, for some, their bioactive peptide derivatives (“matrikines”) as mediators of cardiovascular cell function [9-12]. Despite major improvements in our understanding of non-fibrillar collagens in cardiac biology, many of the groups that make up this family have remained underexplored. One such group includes the Fibril-Associated Collagens with Interrupted Triple helices (FACITs). The FACITs include eight distinct members, specifically, collagens type IX, XII, XIV, XVI, XIX, XX, XXI, and XXII. Of these, six display transient and/or sustained expression in the mammalian heart, including types IX [13], XII [14], XIV [15], XVI [16], XXI [17], and XXII [18]. Though knowledge of their function in the heart is considerably limited, reports of the heightened expression of a number of FACITs during cardiac development (e. g., types IX [13], XIV [15], XVI [16]), as well as injury (e.g., XII [19], XIV [20], XVI [8], XXI [8]) suggest potentially important biological roles for these low abundance collagens. Though detailed insights regarding the function of FACITs is limited, they may influence cardiomyocyte size, myocardial organization, and ventricular function. For instance, collagen XIV (encoded by *Col14a1*) null mice exhibit altered production of other collagen types and remodeling enzymes, disorganized ventricular collagen fibrils, diminished ventricular performance, and reduced cardiomyocyte growth [15]. Such insights support the premise that FACITs could be important arbiters of cardiomyocyte function. This also raises questions as to whether other members of this group share redundant or distinct biological roles in such processes.

Though a majority of collagen types that make up the FACIT family have confirmed cardiac localization in the mammalian heart, the distribution of one particular FACIT, type XIX, has remained controversial. For example, an early study surveying the expression patterns of collagen type XIX found that its mRNA was ubiquitously expressed in tissues during mouse embryogenesis (limbs, vertebrae, heart, brain, tail, kidneys, calvaria, lung, muscle, skin, and intestine), but its expression became constrained to only a few non-cardiac tissues in adulthood [21]. Conversely, recent studies have detected collagen XIX in both whole tissue RNA extracts [22] and mesenchyme protein isolates [23] from adult murine hearts. There are no other reports on the biological importance of collagen XIX in the heart, except the recent discovery that it is elevated upon cardiac injury [22]. Perhaps due to its exceptionally scarce abundance in the ECM (≈10^−6^% of dry tissue weight) [24] and limited reports of its detection in adult myocardium, the biological significance of collagen XIX has gone unnoticed. Here, we addressed this gap in knowledge by examining the impact of *Col19a1* deletion on cardiac structure and function.

## Results

### Fibroblasts are a major source of collagen XIX in the adult mammalian heart

**Fig. 1.**
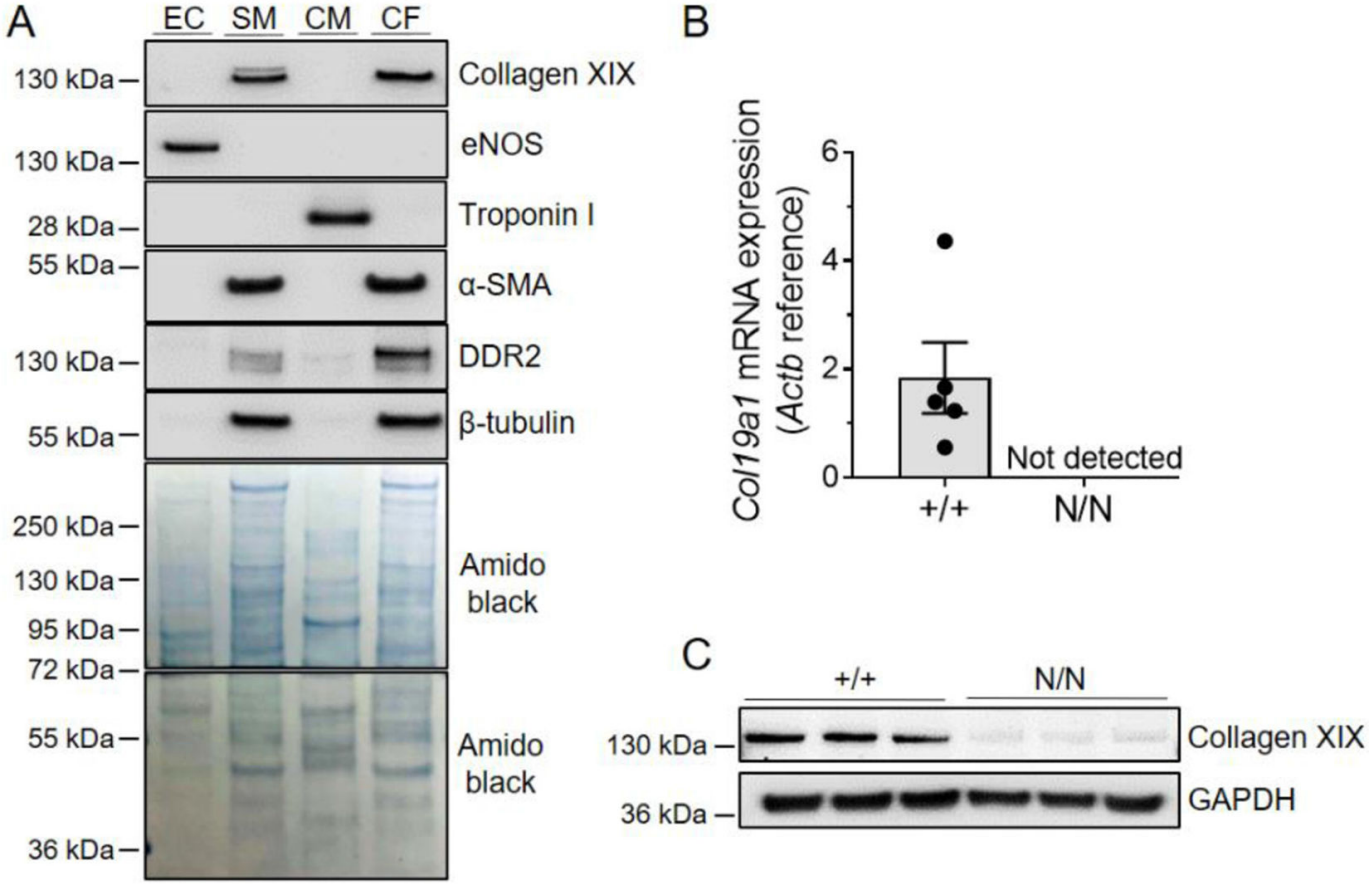
Cardiac fibroblasts express collagen XIX. **A** Representative immunoblots using total protein extracts sourced from different cardiovascular cell types to evaluate collagen XIX protein expression levels. Cellular expression patterns and enrichment levels of key protein markers were used to distinguish among coronary endothelial (EC: eNOS^+^), coronary smooth muscle (SM: αSMA^+^, DDR2^high^), cardiomyocyte (CM: troponin I), and cardiac fibroblast (CF: αSMA^+^, DDR2^low^) cells. Amido black was used as a loading control. **B** RT-PCR (+/+, *N* = 5; N/N, *N* = 6) and **C** Western blot analysis (+/+, *N* = 3; N/N, *N* = 3) of collagen XIX expression in cardiac fibroblasts derived from *Col19a1* wildtype (+/+) and null (N/N) mice. Immunoblot markers denote molecular weight standards (in kDa) resolved with experimental protein lysates.

Though collagen type XIX is generally recognized as an extra-cardiac collagen that is not abundant in the adult mammalian heart [21], *Col19a1* mRNA and its encoded alpha-1 chain protein were detected in separate studies using total RNA from adult mouse hearts [22] and protein extracts derived from isolated cardiac fibroblasts [23]. To gain a more complete view of the potential source of collagen XIX in the murine heart, collagen XIX immunoblots were performed using total protein lysates derived from fractionated cardiac cell types, which included endothelium, smooth muscle, ventricular myocytes, and fibroblasts (Fig. 1A). Immunoblots showed that collagen XIX is expressed in both cardiac fibroblasts and coronary smooth muscle, but absent in cardiomyocytes and endothelium (Fig. 1A). Anti-collagen XIX antibody specificity was confirmed in accompanying immunoblots utilizing *Col19a1*^*N/N*^ cardiac fibroblasts, in which neither *Col19a1* mRNA nor protein was produced (Fig. 1B,C). Examination of *Col19a1* mRNA and protein expression in wildtype cardiac fibroblasts revealed transcript levels to be of relatively low abundance, with mean cycle threshold (Ct) values of ≈32 in rT-PCR analyses (Fig. 1B). Yet, collagen XIX protein was consistently detected in wildtype cardiac fibroblasts via immunoblot (Fig. 1C). These data suggest that cardiac fibroblasts and smooth muscle cells are the predominant source of collagen XIX in the adult mammalian heart—a predicted observation as both cell types are considered the chief producers of ECM components in cardiac tissue [25].

### *Col19a1* nullizygous mice display reduced systolic function and ventricular remodeling

**Fig. 2.**
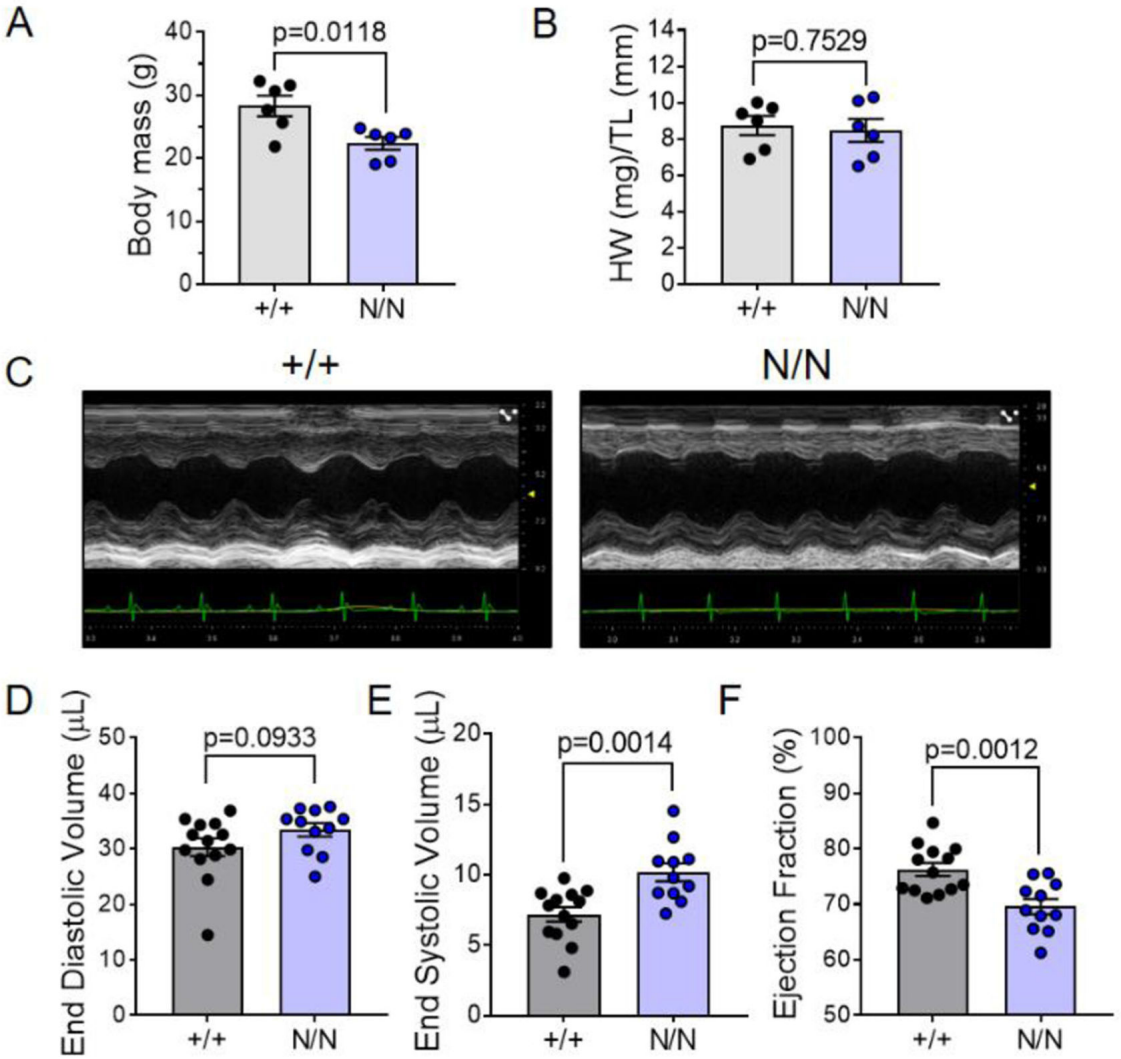
*Col19a1* null mice display reduced systolic function. *Col19a1* wildtype (+/+) and null (N/N) mice subjected to gravitometric (**A** body mass and **B** heart weight:tibia length ratio; +/+, *N* = 6 [2 female, 4 male] and N/N, *N* = 6 [2 female, 4 male]) and echocardiographic assessment (**C** representative M-mode, **D**–**E** ventricular volumes, and **F** ejection fraction; +/+, *N* = 13 [7 female, 6 male] and N/N, *N* = 11 [9 female, 2 male]) at 18–20 wk of age. Volume data were calculated from 2D long axis measurements. Graphs depict arithmetic mean ± SEM. Statistics: All data were subject to the Shapiro–Wilk test for normality. **D** was analyzed using the non-parametric Mann–Whitney U test. **E**–**F** were analyzed by an unpaired, two-tailed Student’s T-test. *p* < 0.05 was considered statistically significant.

To uncover the biological significance and/or potential functional roles for this non-fibrillar collagen in the heart, collagen XIX nullizygous mice (*Col19a1*^*N/N*^, denoted as “N/N”) were examined for evidence of underlying cardiovascular structural and/or functional phenotypes. Initial examination of *Col19a1* null animals at 18–20 weeks of age revealed minor decreases in body mass relative to wildtype (*Col19a1*^*+/+*^, denoted as “+/+”) littermates (Fig. 2A). Changes in body weight were not accompanied with differences in heart mass to tibia length quotients (Fig. 2B). Blinded echocardiographic analyses were employed to evaluate the consequences of *Col19a1* ablation on postnatal ventricular volumes, dimensions, and cardiac performance. Volume analyses showed no difference in left ventricular end diastolic volumes (EDV) between *Col19a1* null and littermate controls; however, they did reveal modest, yet statistically significant increases in left ventricular end systolic volumes (ESV) in *Col19a1* nullizygous animals (Fig. 2C-E). Despite said volume differences, cardiac stroke volumes remained comparable between *Col19a1* null and wildtype animals (Table 1). Further, as there were no changes in heart rate between *Col19a1* null and wildtype animals (Table 1), cardiac output remained comparable (Table 1). Indices of cardiac function, including fractional shortening (measured from short axis M-mode echocardiograms) and left ventricular ejection fraction (measured from parasternal long axis B-mode echocardiograms), revealed decrements in systolic function in *Col19a1* null animals compared to wildtype (Fig. 2F and Table 1). *Col19a1* null animals also showed evidence of mild left ventricular remodeling (Table 1). End diastolic left ventricular internal diameters were similar among groups; however, end systolic internal diameters were larger in *Col19a1* null animals, compared to wildtype (Table 1). Accompanying these changes in luminal diameter, *Col19a1* null animals displayed statistically significant reductions in both left ventricular anterior and posterior wall thickness in end diastole and systole (Table 1), relative to littermate controls. Together, these data suggest a novel role for collagen XIX in cardiac structure and function.

### Cardiomyocyte hypertrophy, but not overt fibrosis, in collagen XIX-deficient mice

**Fig. 3.**
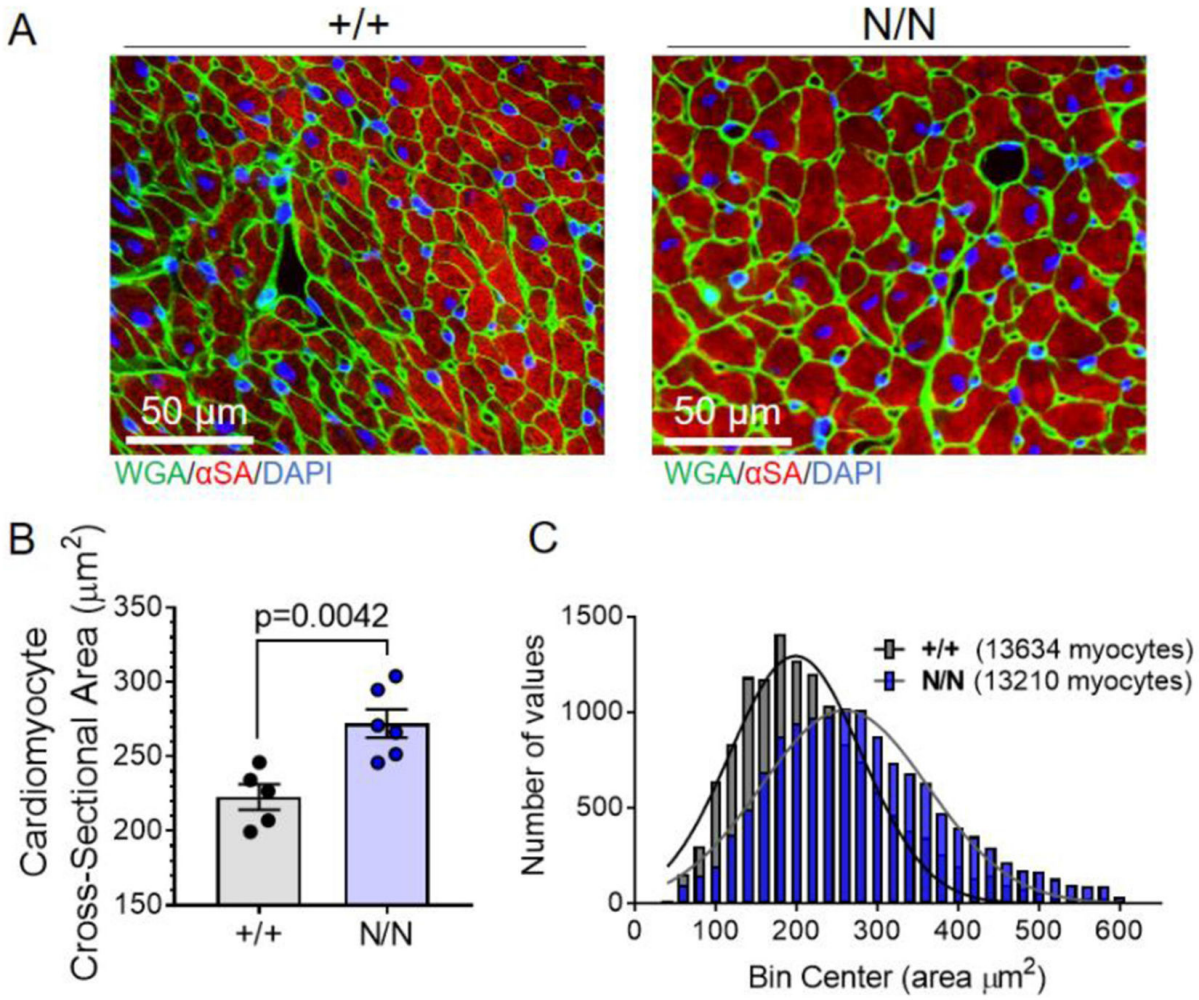
*Col19a1* deficient mice exhibit increased cardiomyocyte size. **A** Representative myocardial tissue sections derived from *Col19a1* wildtype (+/+) and null (N/N) mice. Sections stained with antibodies against α-sarcomeric actin (α-SA; myocytes; red) and wheat germ agglutinin (WGA; cell membranes; green). Nuclei counterstained with DAPI (nuclei; blue). **B** Graphs illustrate mean cardiomyocyte cross-sectional area ± SEM in wildtype (+/+; *N* = 5 [2 female, 3 male]) and null (N/N; *N* = 6 [2 female, 4 male]) mice. **C** Accompanying histograms showing cardiomyocyte size distribution (number of myocytes vs. cardiomyocyte size [bin center; area]) in +/+ and N/N animals. Bin width=20. Inset legend reports total number of myocytes measured per group. Statistics: Data normality was assessed using the Shapiro–Wilk test. Data in **B** was analyzed by an unpaired, two-tailed Student’s T-test. *p* < 0.05 was considered statistically significant.

**Fig. 4.**
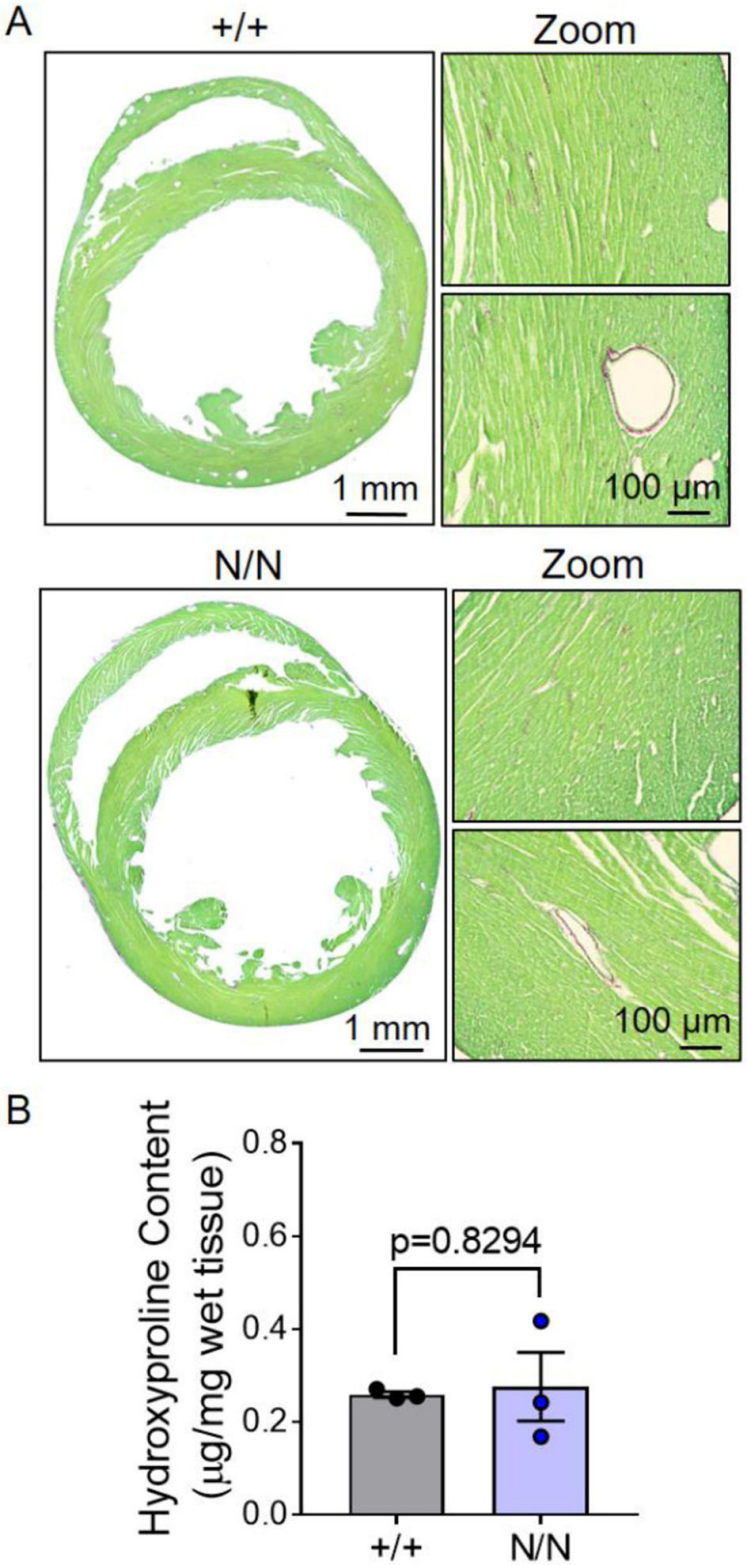
Myocardial collagen content is similar among *Col19a1* null and wildtype mice. **A** RepresentaW *Col19a1* wildtype (+/+, *N* = 3) and null (N/N, *N* = 3) mice stained with Sirius Red (stains [Gly-X-Y]_n_ of fibrillar collagens; red) and Fast Green (stains non-collagenous proteins; green). **B** Myocardial hydroxyproline content in +/+ (*N* = 3 [2 female, 1 male]) and N/N (*N* = 3 [2 female, 1 male]) mice. Graphs depict arithmetic mean ± SEM. Statistics: Data normality was assessed using the Shapiro–Wilk test. Data in **B** was analyzed by an unpaired, two-tailed Student’s T-test. *p* < 0.05 was considered statistically significant.

Myocardial tissue sections were stained and evaluated for cardiomyocyte cross-sectional area in 18–20-week-old *Col19a1* null and wildtype littermate mice (Fig. 3). Automated computational analyses were performed without regional bias on transverse, mid-ventricular tissue sections derived from both wildtype and *Col19a1* null mice (Fig. 3A). Analyses revealed that cardiomyocyte cross-sectional areas were significantly greater in *Col19a1* null mice compared to controls (Fig. 3B). Further, accompanying histograms displaying the size distribution of the more than 13,000 cardiomyocytes measured in each group revealed a discernible shift in the abundance of smaller cardiomyocytes (<250 μm^2^) to larger cardiomyocytes (>250 μm^2^) in *Col19a1* null hearts relative to wildtype (Fig. 3C). This suggests collagen XIX influences cardiomyocyte growth and/or maturation, but the mechanism is unknown. Because this particular FACIT [26] and others [27] may regulate collagen fibril abundance, organization, and stability, alterations in ECM dynamics may be one mechanism by which collagen XIX exerts its biological influence on cardiac growth and structure. To this end, myocardial collagen abundance was evaluated in hearts of 18–20-week-old mice (Fig. 4). Transverse myocardial tissue sections stained with picrosirius red/fast green (Fig. 4A) revealed no apparent differences in myocardial collagen content among collagen XIX-deficient and littermate control animals. Further supporting these observations, accompanying hydroxyproline assays utilizing cardiac tissue homogenates also failed to detect any alterations in total myocardial collagen levels between *Col19a1* null and wildtype (Fig. 4B)—excluding bulk fibrosis as a contributor to the cardiovascular phenotypes in *Col19a1* null mice.

Because collagen XIX is considered a basement membrane (BM) associated protein [24], transverse myocardial tissue sections were stained for nidogen 1 to ascertain the impact of collagen XIX deletion on myocardial basement membrane morphology (Supplementary Fig. S1). Compared to wildtype, *Col19a1* null hearts showed no discernable irregularities in basement membrane zone morphology (Supplementary Fig. S1). In addition, there was no evidence of basement membrane expansion in intracellular regions or alterations in staining intensity (Supplementary Fig. S1). Overall, these data indicate that the cardiomyocyte hypertrophy observed in *Col19a1* null animals is neither connected to perturbations in basement membrane assembly nor interstitial collagen accumulation.

### Collagen XIX regulates cardiac fibroblast collagen synthetic and stabilization enzyme expression

**Fig. 5.**
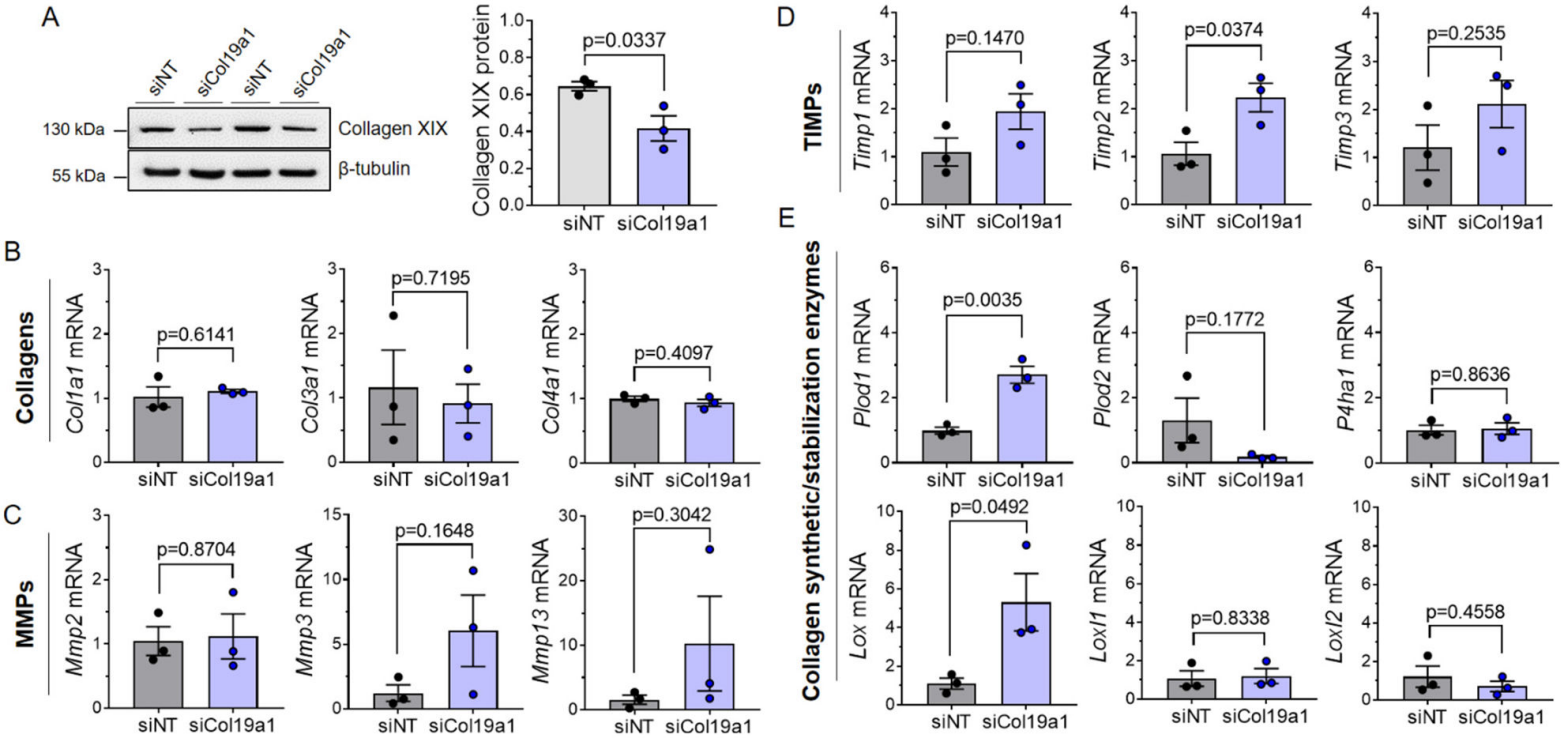
siRNA-mediated depletion of collagen XIX in cardiac fibroblasts alters expression of collagen synthetic/stabilization enzymes. **A** Immunoblots quantifying collagen XIX protein expression in wildtype cardiac fibroblasts transfected with Col19a1 siRNA (siCol19a1) or non-target control (siNT). β-tubulin is a loading control. Graphs report mean collagen XIX protein expression (relative to β-tubulin loading controls) ± SEM. Immunoblot markers denote molecular weight standards (in kDa) resolved with experimental protein lysates. Gene expression assays employing transcript-specific primers targeting **B** collagens (*Col1a1, Col3a1*, and *Col4a1*), **C** matrix metalloproteinases (*Mmp2, Mmp3, Mmp13*), **D** tissue inhibitors of metalloproteinases (*Timp1, Timp2, Timp3*), and **E** collagen synthetic (*Plod1, Plod2, P4ha1*)/stabilization enzymes (*Lox, Loxl1, Loxl2*). Graphs report mean mRNA expression (relative to siNT controls) ± SEM. *Gapdh* was used as an internal reference control. All experiments were performed using three biological replicates (*N* = 3 independent cardiac fibroblast isolations [2 female, 1 male]), with each cell line performed in triplicate. Statistics: Data normality was assessed using the Shapiro–Wilk test. Data were analyzed by an unpaired, two-tailed Student’s T-test. *p* < 0.05 was considered statistically significant.

**Fig. 6.**
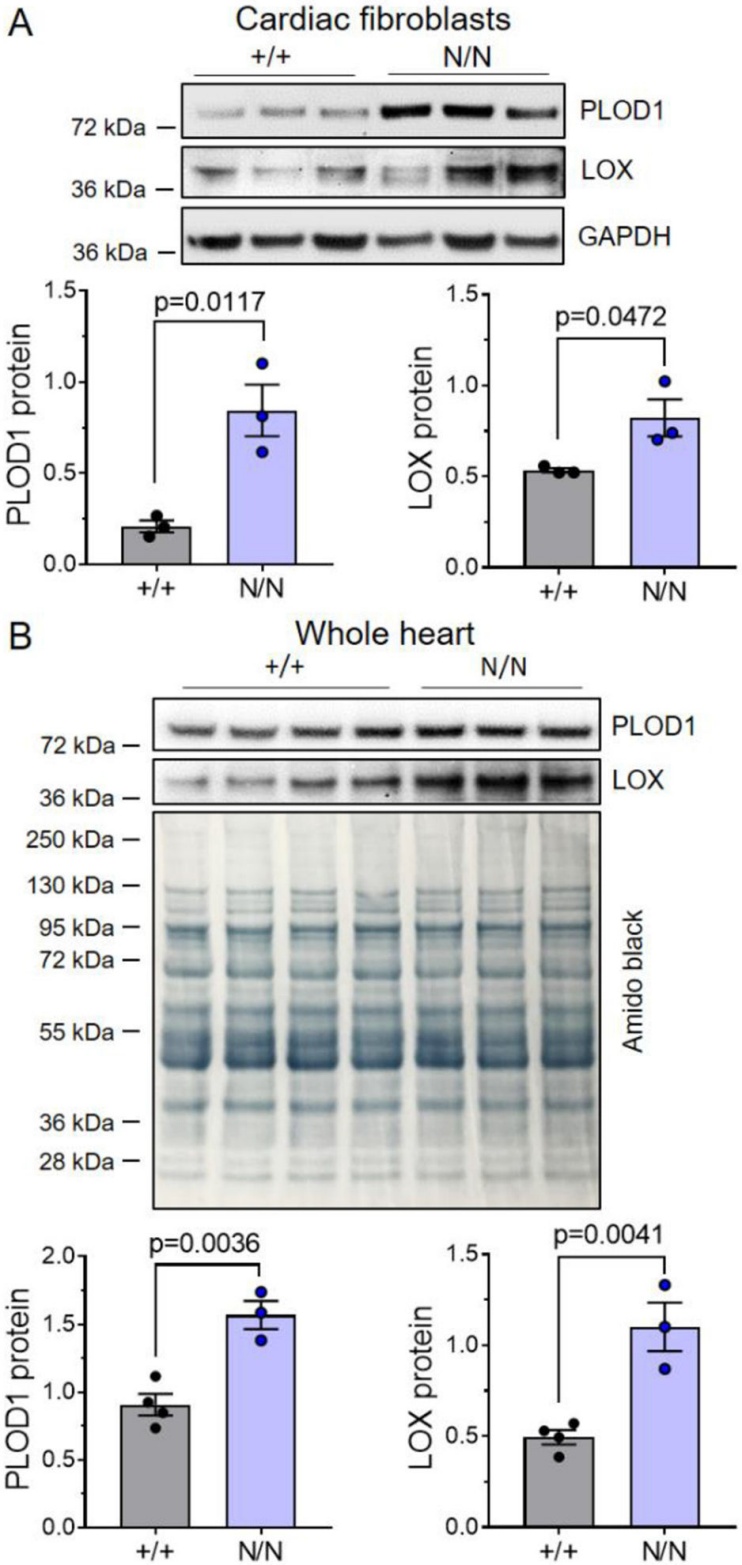
Collagen XIX regulates the expression of enzymes involved in collagen synthesis and stability. Western analyses of PLOD1 and LOX enzyme expression in **A** cardiac fibroblasts and **B** hearts derived from *Col19a1* wildtype (+/+) and null (N/N) mice. A Representative PLOD1/LOX immunoblots utilizing cardiac fibroblast extracts (*N* = 3 independent isolations per genotype [1 female, 2 male]). GaPdH is a loading control. **B** PLOD1/LOX immunoblots using whole heart lysates (+/+, *N* = 4 [2 female, 2 male]; N/N, *N* = 3 [1 female, 2 male]). Amido black is a loading control. Immunoblot markers denote molecular weight standards (in kDa) resolved with experimental protein lysates. Graphs report mean protein expression (relative to loading control) ± SEM. Statistics: Data normality was assessed using the Shapiro–Wilk test. Data were analyzed by an unpaired, two-tailed Student’s T-test. *p* < 0.05 was considered statistically significant.

Reports that FACITs can participate in the modulation of ECM dynamics [15,26,27] led us to test whether alterations in ECM architecture may underlie structural differences in collagen XIX deficient animals. Because fibroblasts are a source of collagen XIX in the heart (Fig. 1), we next examined the effects of collagen XIX depletion on the expression of structural collagens (*Col1a1, Col3a1*, and *Col4a1*) and ECM remodeling enzymes (MMPs, TIMPs, LOXs, and PLODs) in isolated cardiac fibroblasts (Fig. 5). In agreement with our histological analyses of *Col19a1* nullizygous mice, which showed no evidence of myocardial fibrosis, siRNA-mediated depletion of *Col19a1* had no effect on mRNA levels of key structural collagens (*Col1a1, Col3a1*, and *Col4a1*), relative to non-target-transduced controls (siNT) (Fig. 5A,B). Though collagen transcripts remained unchanged, a number of enzymes responsible for ECM turnover (e.g., *Timp2*) and the normal assembly and cross-linking of collagen fibrils (i.e., *Plod1* and *Lox*), were differentially expressed in si*Col19a1*-transduced fibroblasts relative to siNT controls (Fig. 5C-E). Of these, *Plod1, Lox*, and *Timp2* mRNA levels were significantly elevated in si*Col19a1*-transduced fibroblasts compared to controls. Congruent with these observations, the expression of PLOD1 and LOX proteins were markedly elevated in both isolated cardiac fibroblasts (Fig. 6A) and in whole hearts (Fig. 6B) of *Col19a1* null mice. These results indicate that collagen XIX does not directly impact cardiac mesenchyme collagen production itself, but in fact modulates the expression of key enzymes involved in their synthesis and stabilization—suggesting potential roles for this FACIT in the regulation of fibrillar collagen assembly and structure.

### Collagen XIX modulates ECM fibrillar collagen structure/organization

**Fig. 7.**
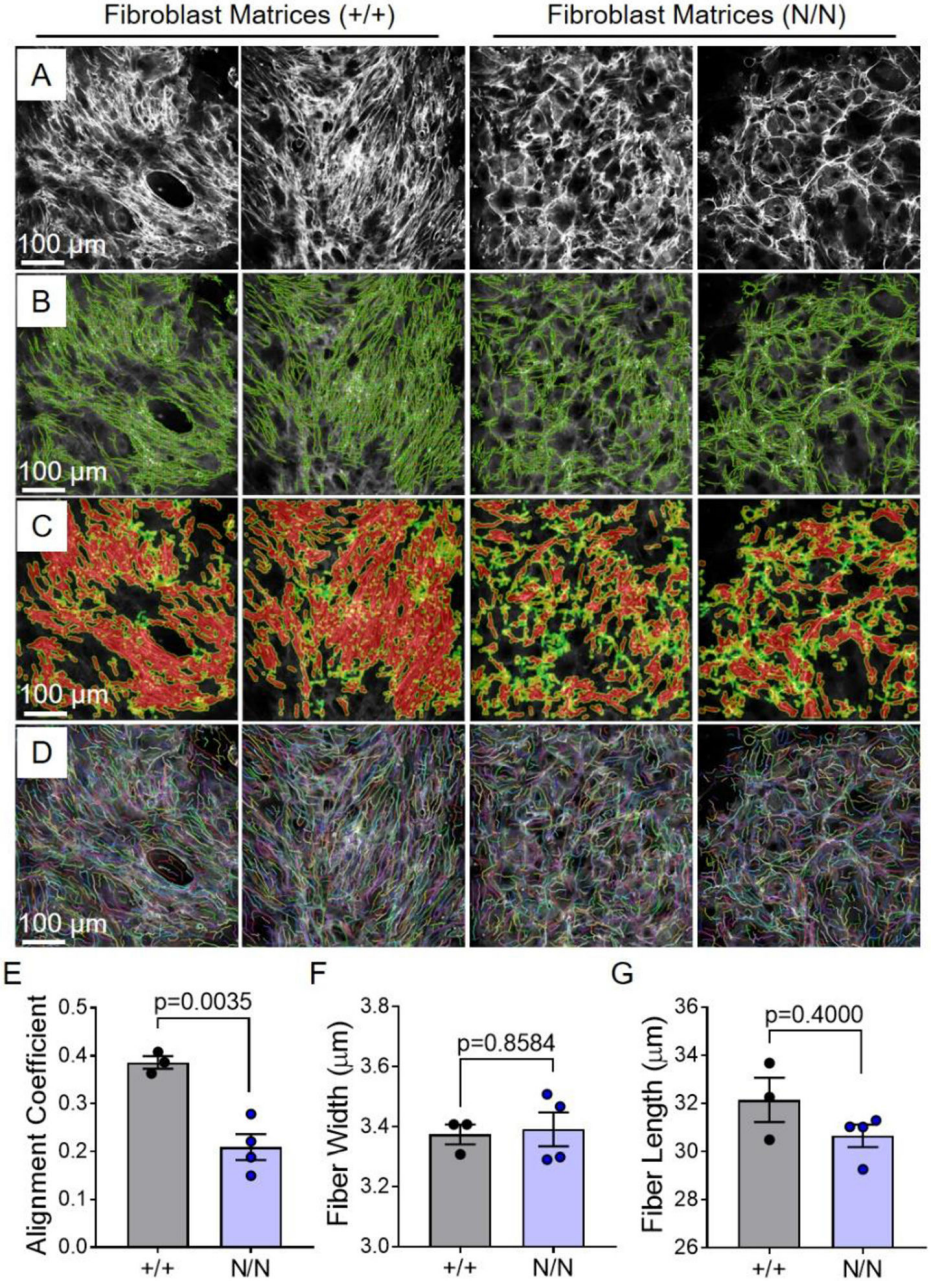
*Col19a1* null fibroblast-derived ECM exhibits reduced collagen organization. Cardiac fibroblasts isolated from *Col19a1* wildtype (+/+) and null (N/N) mice were grown to confluence for 7 d **A** Resultant collagen matrices were imaged via second harmonic generation microscopy (SHG; Nikon A1R MP+ multiphoton). Fibrillar collagen alignment was quantified using CurveAlign/CT-FIRE. **B** Image generated by CurveAlign showing the location (red dots) and orientation (green lines) of extracted fibers. **C** Heatmap indicating regional fiber alignment (red denotes areas of greater alignment). **D** CT-FIRE extracted fibers overlaid on master image (A). Information for entire fibers are shown and highlighted by different colored lines for contrast. Graphs illustrate mean fibrillar collagen **E** alignment, **F** width, and **G** length ± SEM in wildtype (+/+; *N* = 3 independent fibroblast isolations [3 female]) and null (N/N; *N* = 4 independent fibroblast isolations [2 female, 2 male]). Statistics: Data normality was assessed using the Shapiro–Wilk test. Data in **E** were analyzed by an unpaired, two-tailed Student’s T-test. Data in **F**–**G** were analyzed using the non-parametric Mann–Whitney U test. *p* < 0.05 was considered statistically significant.

**Fig. 8.**
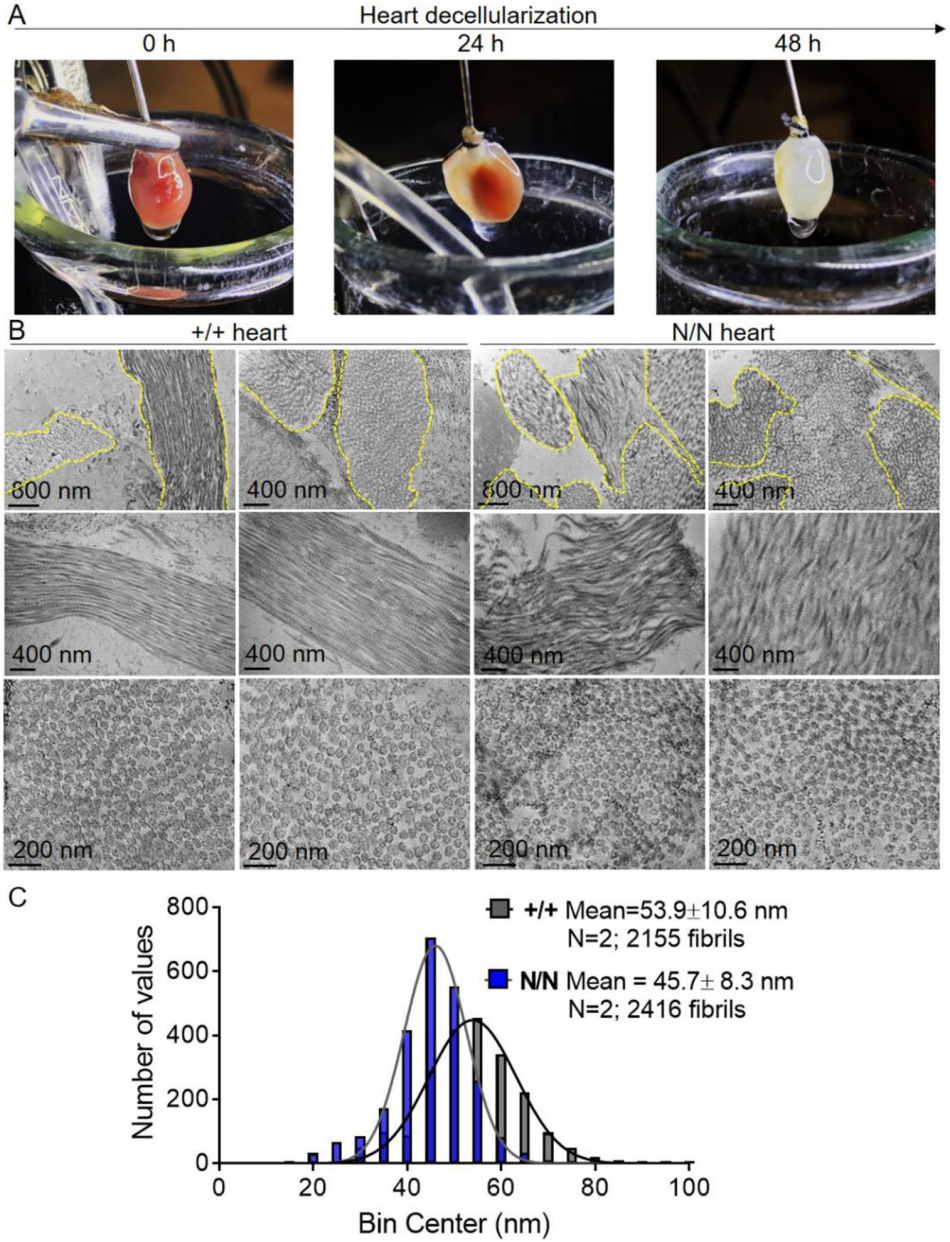
Col19a1 null mice display decreased myocardial collagen fiber organization and reduced fibril diameter. Hearts sourced from *Col19a1* wildtype (+/+, *N* = 2 [1 female, 1 male]) and null (N/N, *N* = 2 [1 female, 1 male]) mice at 18–20 wk were subjected to **A** detergent-based decellularization procedures for 48 h. Decellularized specimens were analyzed via **B** transmission electron microscopy. Electron micrographs reveal significant heterogeneity in size and spatial organization of individual collagen fibers (top row; outlined in yellow) in *Col19a1* nulls compared to wildtype. Longitudinal (middle row) and transverse (bottom row) morphology of collagen fibrils reveal abnormal collagen fibril packing, reduced fibril alignment, and decreased fibril diameters in nulls relative to wildtype. **C** Accompanying histograms showing collagen fibril diameter distribution (number of fibrils vs. diameter [bin center; nm]) in +/+ and N/N animals. Bin width=5. Inset legend reports mean fibril diameter ± SD and total number of fibrils measured per group.

PLOD1 catalyzes hydroxylation of lysine residues in collagen alpha chains (critical for the stability of intermolecular crosslinks) [28-31], and LOX participates in the oxidative deamination of lysine/hydroxylysine residues (important for the formation of covalent pyridinoline cross-links between individual collagen fibers and stabilization of heterotypic fibrils) [32-34]. Hence, we hypothesized that collagen XIX may participate in the regulation of cardiac fibroblast collagen assembly/organization. To this end, fibrillar collagen structure/organization was evaluated in matrices derived from *Col19a1* null and wildtype fibroblasts in culture (Fig. 7). Fibroblast-produced matrices were imaged by second harmonic generation (SHG) microscopy and subsequently subjected to quantification. There, imaged collagen fibers were digitally extracted, processed, and quantified (Fig. 7A-D); providing values pertaining to ECM collagen fiber alignment, fiber width, and fiber length (Fig. 7E-G). While there were no discernible differences in cell morphology among these populations of fibroblasts (Supplementary Fig. S2), matrices produced from *Col19a1* null cells exhibited pronounced reductions (≈46%) in CurveAlign program-computed alignment coefficients compared to wildtype controls (Fig. 7E). Though alterations in ECM organization were observed, CT-FIRE program dimensional measurements of extracted fibers revealed no changes in collagen fiber widths or lengths among matrices sourced from *Col19a1* null and wildtype fibroblasts (Fig. 7F,G). In addition, transmission electron micrographs of decellularized mouse hearts (Fig. 8A) revealed pronounced alterations in the spatial organization and morphology of collagen fibers in *Col19a1* null hearts compared to wildtype animals. Specifically, *Col19a1* null hearts exhibited significant heterogeneity in both the shape and size of individual collagen fibers (Fig. 8B, top row). Assessment of longitudinal-imaged fibers in *Col19a1* null hearts showed evidence of abnormal packing and reduced alignment of individual collagen fibrils (Fig. 8B, middle row). In addition to these structural abnormalities, inspection of transverse-imaged fibers uncovered reductions in collagen fibril diameters in null animals relative to wildtype littermates (Fig. 8B, bottom row). These results, tabulated in accompanying histograms incorporating the dimensions of the more than 4000 individually measured collagen fibrils, showed a near 15% decrease in mean fibril diameter in *Col19a1* nulls relative to their wildtype counterparts (Fig. 8C). Collectively, these data indicate that collagen XIX has important functions in ECM fibrillar collagen structure/organization in the mammalian heart.

### De-repression of the focal adhesion kinase signaling pathway accompanies cardiomyocyte hypertrophic growth in *Col19a1* nullizygous animals

**Fig. 9.**
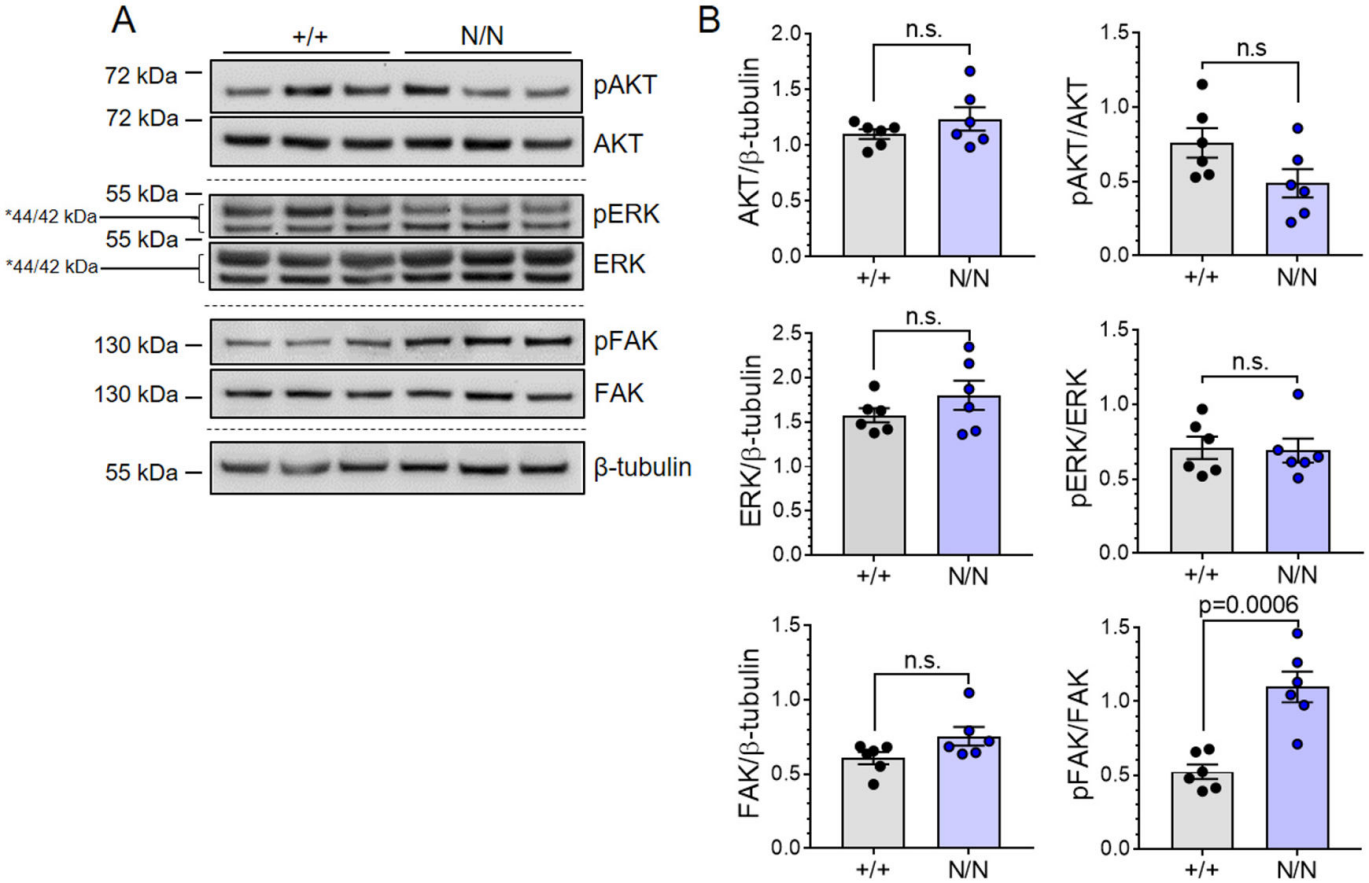
*Col19a1* deficiency results in enhanced non-canonical FAK signaling. **A** Representative immunoblots assessing the expression of phosphorylated and total AKT, ERK, and FAK proteins in myocardial tissues sourced from *Col19a1* wildtype (+/+) and null (N/N) mice at 18–20 wk. β-tubulin is a loading control. Immunoblot markers denote molecular weight standards (in kDa) resolved with experimental protein lysates. Those with asterisks (*) indicate predicted molecular weights of visualized protein targets. **B** Graphs report total kinase levels relative to β-tubulin ± SEM (left column) and total phosphorylated kinase levels relative to total kinase protein ± SEM (right column). *N* = 6 independent biological replicates per group [3 female, 3 male]. Statistics: Data normality was assessed using the Shapiro–Wilk test. pERK/ERK immunoblots data were analyzed by an unpaired, two-tailed Student’s T-test. pAKT/AKT and pFAK/FAK data were analyzed using the non-parametric Mann–Whitney U test. *p* < 0.05 was considered statistically significant.

**Fig. 10.**
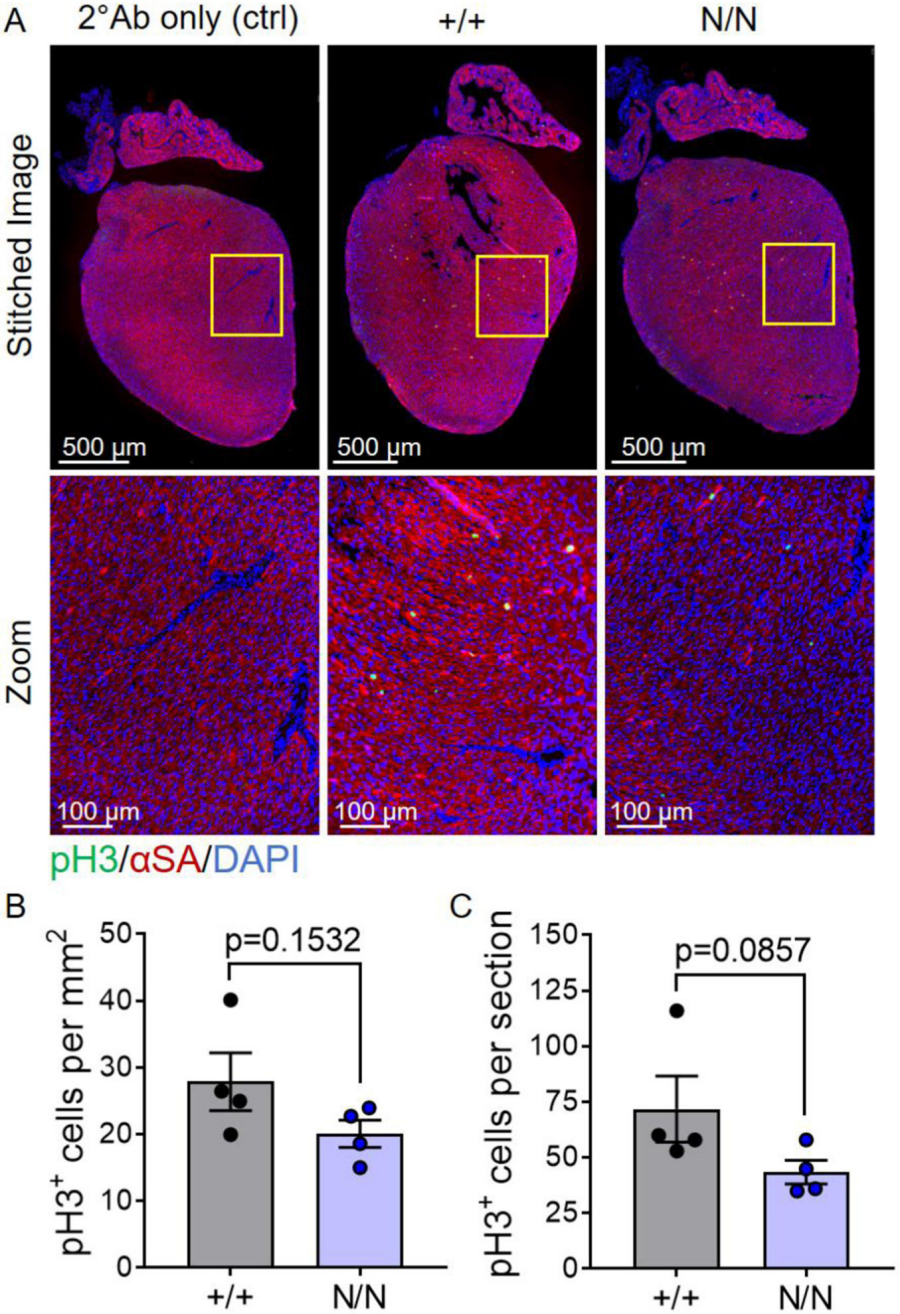
Postnatal cardiomyocyte proliferation is conserved in *Col19a1* null mice. **A** Coronal myocardial tissue sections from *Col19a1* wildtype (+/+) and null (N/N) mice at postnatal day 3. Sections were stained with antibodies against phospho-histone H3 (pH3; proliferation marker; green) and α-sarcomeric actin (αSA; myocytes; red). Nuclei were counterstained with DAPI (nuclei; blue). Sections incubated with only secondary antibodies [2° Ab (ctrl)], in the absence of anti-pH3 antibody, were included as negative staining controls. Graphs depict the mean number of pH3^+^ cells per **B** unit area (mm^2^) ± SEM or **C** tissue section ± SEM in wildtype (+/+; *N* = 4) and null (N/N; *N* = 4) neonates. Statistics: Data normality was assessed using the Shapiro–Wilk test. Data in **B** was analyzed by an unpaired, two-tailed Student’s T-test and data in **C** by the non-parametric Mann–Whitney U test. *p* < 0.05 was considered statistically significant.

**Fig. 11.**
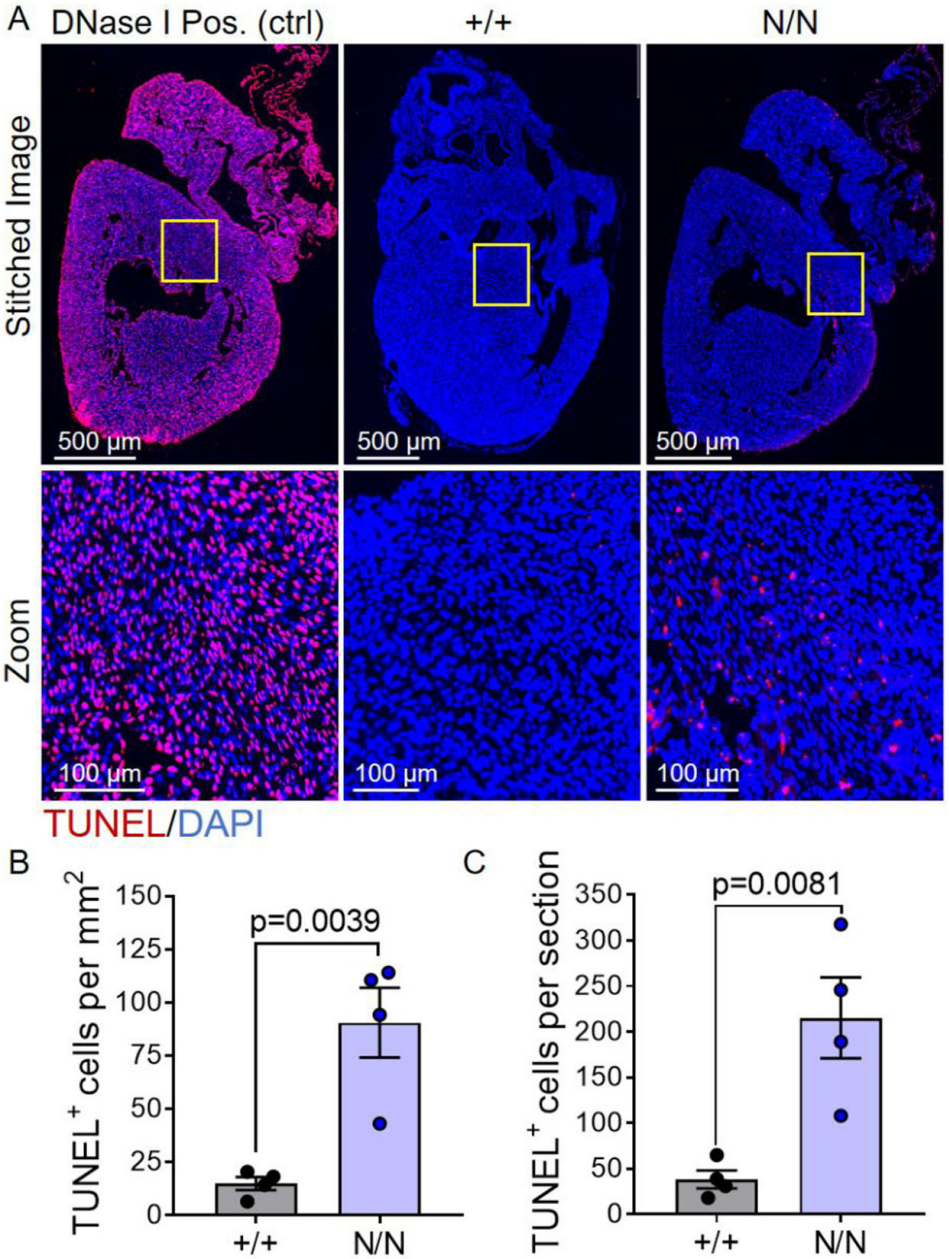
Col19a1 deficient mice display elevated cellular apoptosis during the first week of postnatal cardiac development. **A** Coronal myocardial tissue sections from *Col19a1* wildtype (+/+) and null (N/N) mice at postnatal day 3. Sections were subjected to TUNEL (terminal deoxynucleotidyl transferase dUTP nick-end labeling; red) staining. Nuclei were counterstained with DAPI (nuclei; blue). Dnase I treated sections served as positive controls [Dnase I Pos. (ctrl)] for TUNEL staining. Graphs depict the mean number of TUNEL^+^ cells per **B** unit area (mm^2^) ± SEM or **C** tissue section ± SEM in wildtype (+/+; *N* = 4) and null (N/N; *N* = 4) neonates. Statistics: Data normality was assessed using the Shapiro–Wilk test. Data in **B** and **C** were analyzed by an unpaired, two-tailed Student’s T-test. *p* < 0.05 was considered statistically significant.

The biological underpinnings of the cardiovascular phenotypes manifested (reduced systolic function and increased cardiomyocyte size) in *Col19a1* null mice are not immediately clear; however, because ECM-dependent integrin signaling influences cardiomyocyte proliferation and hypertrophy [35], we posited that perturbation in ECM structure could contribute to cardiomyocyte hypertrophy observed in collagen XIX knockout animals. To begin to address this idea, analyses of the activation of canonical downstream integrin receptor pathways (i.e., PI3K/AKT, MEK/ERK, and SRC/FAK) were evaluated in *Col19a1* null and wildtype mouse hearts (Fig. 9). Immunoblots revealed no significant change in the expression levels of phosphorylated AKT (pAKT) or ErK (pERK) among wildtype and mutant hearts (Fig. 9); however, a significant increase in pFAK was observed in *Col19a1* null animals (Fig. 9). Elevated pFAK levels in *Col19a1* null animals were not associated with increased myocardial expression of the dominant cardiomyocyte β integrin, specifically integrin β1 (Supplementary Fig. S3), which comprises the integral β subunit component of the major heterodimeric binding receptors for collagen (α1β1), fibronectin (α5β1), and laminin (α7β1) on cardiomyocytes [36]. These results suggest that the loss of *Col19a1* results in de-repression of the focal adhesion kinase pathway—a pathway that is central for the development of load-induced [37] and compensatory [38] cardiomyocyte hypertrophy. While such data implicate enhanced fAk signaling, independent of changes in integrin β1 expression, as a potential molecular pathway underlying this hypertrophic response, primary stimuli driving these phenomena in global *Col19a1* nullizygous in adult animals are undefined. Given that the cardiac ECM itself is a critical regulator of cellular processes in both organ development and homeostasis, we believe the contributing mechanisms may also be attributed to alterations in ECM-dependent signaling influencing cardiomyocyte growth and/or survival during early postnatal cardiac development in collagen XIX knockout animals. To address this, neonatal cardiomyocyte proliferation was comparatively assessed in *Col19a1* null and wildtype mouse hearts at postnatal day 3 via immunohistochemical detection of phospho-histone H3 (pH3) (Fig. 10). Collagen XIX-deficient neonatal hearts displayed conservation of cardiomyocyte proliferation with both groups exhibiting comparable numbers of pH3^+^myocytes per unit area (Fig. 10B) and per myocardial tissue section (Fig. 10C). In addition, myocardial apoptosis was evaluated in neonatal hearts by *in situ* terminal deoxynucleotidyl transferase dUTP nick-end labeling (TUNEL) (Fig. 11). Relative to their wildtype counterparts, neonatal *Col19a1* nullizygous hearts exhibited an approximate 6-fold increase in both the number of apoptotic cells per unit area (Fig. 11B) and per myocardial tissue section (Fig. 11C). Collectively, these findings suggest that apoptosis may contribute to compensatory hypertrophic growth of surviving cardiomyocytes in collagen XIX knockout animals.

Overall, these data provide evidence that fibroblast-derived collagen XIX plays a role in the regulation of cardiac structure and function—and that it may do so via modifying ECM collagen fiber organization. These data also suggest that collagen XIX may regulate ECM superstructure via acting in a juxtacrine fashion to regulate the expression of genes involved in collagen synthesis/stability in cardiac fibroblasts.

## Discussion

Since the earliest studies of the cardiac extracellular matrix nearly four decades ago [1], the field has made significant progress in both clarifying matrix compositional complexity and uncovering the role of its various fibrillar and non-fibrillar collagen components in homeostasis and disease [6]. Nevertheless, our knowledge concerning the biological function of many of its individual components remains limited. We have developed a long-range program to interrogate the specific involvement of heretofore underexplored regulators of the cardiac ECM, including distinct groups of non-fibrillar collagens, such as the FACIT family members. FACITs play fundamental roles in ensuring the stability of the extracellular matrix and its fibrillar collagen network, through regulating the polymerization and size of fibrillar collagens [39] and directing their structural arrangement [15]. Herein, we addressed the role of collagen XIX in shaping the cardiac ECM and, ultimately, the structure and function of the heart. Through the use of *Col19a1* nullizygous animals, we revealed that collagen XIX guides the alignment of the stromal-derived filamentous matrix, which provides structural and spatial context for parenchymal cells within the mammalian heart. *Col19a1* null animals exhibited alterations in the expression of collagen synthetic/stabilization enzymes and disorganization of ventricular collagen fibrils. These findings were accompanied by thinning of the myocardial wall, expansion of cardiomyocyte cross-sectional area, and reductions in systolic function. Collectively, such insights support the broader concept that FACITs can impact the structure and function of the heart.

Although not generally recognized to be expressed in adult myocardium, we found that type XIX collagen is produced by fibroblasts and smooth muscle cells, which is consistent with expression profiles indicating that this protein localizes to the basement membrane of other tissues [24]. Our expression analyses largely agree with more recent investigations that have detected collagen XIX in whole heart tissue RNA extracts [22] and cardiac mesenchyme protein isolates [23]. Consistent with collagen XIX being a scarcely abundant collagen, accounting for approximately 10^−6^% of dry tissue weight [24], *Col19a1* mRNA transcript and protein levels in isolated cardiac mesenchyme were low—showing late cycle threshold values (average Ct values ≈32) in RT-PCR amplification reactions. Likewise, collagen XIX immunoblots required extended durations of membrane exposure (3–5 min) for adequate visualization. Considering that this FACIT has such low expression levels in cardiac fibroblasts, and that fibroblasts account for less than 25% of the myocardial tissue volume [40], may help to explain why collagen XIX protein is not easily detected using traditional antibody-based approaches. Notwithstanding its low level of expression, targeted reduction of *Col19a1* in cardiac fibroblasts increased expression of collagen synthetic/stabilization enzymes (e.g., PLOD1 and LOX). In addition, *Col19a1* deletion reduced alignment of fibroblast-produced collagen matrices, suggesting that collagen XIX can function to finetune fibroblast matrix-modifying capacity. These observations were mirrored in the hearts of global *Col19a1*^*N/N*^ mice, wherein collagen synthetic/cross-linking enzymes PLOD1 and LOX were elevated and accompanied by myocardial collagen disorganization and smaller fibrils. Although we did not address the degree to which the elevated expression of PLOD1 and LOX enzymes contribute to the observed changes in cardiac ECM architecture or fibril morphology in *Col19a1* nullizygous animals, our finding that PLOD1 expression negatively correlates with fibril diameter is consistent with other studies and the established function of PLOD1 [41].

Our results highlight a potential relationship between FACIT-dependent alterations in matrix geometry and the regulation of myocardial growth and function. And while our data point to collagen XIX as an important regulator in this process, similar roles have been described for another FACIT in the heart. In fact, collagen XIV (*Col14a1*) knockout mice, too, display altered expression of remodeling enzymes, disorganized ventricular collagen fibrils, and reductions in ventricular function [15]; however, contrary to our *Col19a1*^*N/N*^ mice, which show enhancement in cardiomyocyte size, *Col14a1*^*−/−*^ mice exhibit reductions in cardiomyocyte size in postnatal stages [15]. While we have yet to understand the fundamental mechanisms underlying their distinct impacts on cardiac physiology *in vivo* knockout models, such findings highlight potentially diverse roles for these FACIT members in the establishment and/or maintenance of the cardiac ECM. These observations also implicate FACIT-mediated modifications in ECM collagen architecture regulate cardiac structure and function. Though the precise mechanism(s) remain to be fully explicated, we deduce cellular integrins as major effectors in these processes. Cellular integrins play essential roles in cell sensing and adaptation to physical constraints imposed by the ECM. Both ECM spatial organization and ECM mechanical properties can influence cellular integrin-mediated signaling [42,43]. Thus, collagen XIX deletion may contribute to the cardiac phenotypes through ECM architectural-dependent alterations in integrin receptor signaling. If true, such changes could impact cardiomyocyte growth and/or survival, which could result from alterations in myocardial mechanical properties and/or modifications in ECM/myocyte integrin receptor engagement. It is known that RGD- and Col-dependent integrins regulate numerous cardiomyocyte biological processes, including, growth [44], force production [45,46], and cellular apoptosis [47,48]. This may help to explain the conspicuous induction of cellular apoptosis in early neonates and the augmented cardiomyocyte hypertrophic growth observed in adult *Col19a1* nullizygous animals. As the current study utilized conventional global collagen XIX knockout animals, it is difficult to ascertain the extent to which the cardiovascular phenotypes derive from the impact of collagen XIX deficiency on early cardiac development and homeostasis. Nevertheless, the loss of myocardial cells at early postnatal timepoints (due to increased apoptosis) may contribute indirectly to the development of compensatory myocyte hypertrophy in adult *Col19a1* nullizygous animals. Notwithstanding, as the ECM is also a critical regulator of cellular processes in organ homeostasis, we surmise that deficits in cardiac function and alterations in myocyte growth also be credited to perturbations in ECM-dependent signaling influencing cardiomyocyte growth and/or function in adult animals.

Beyond ECM-dependent mechanisms, collagen XIX-derived matrikines themselves exert biological activity. In cancer cells [49] and neurons [50], the non-collagenous C-terminal domain (NC1) of collagen XIX (cleaved by plasmin) can modulate cell responses via binding and signaling through RGD-dependent integrins (including αvβ3 and α5β1). Interestingly, RGD integrin signaling regulates cardiac fibroblast activation [51], cardiomyocyte growth [44], and force production [45,46]. Hence, we speculate that the mechanisms by which collagen XIX regulates cardiac function are manifold and may include both changes in ECM architecture/biomechanics (reducing their engagement with the ECM) and NC1 matrikine-mediated antagonism of cardiomyocyte integrin receptor activity. In light of the extensive evidence showing ECM/integrin receptor signaling as important mediators of cardiomyocyte biology [35,38,45], it is plausible that NC1 matrikine may directly regulate cardiomyocyte growth and/or function through its direct interactions with cardiomyocyte integrins in adult animals. FAK, a major kinase involved in the integrin intracellular signaling cascade, is activated by various growth factors and by ligation of all β1-, β3-, or β5-containing integrins [52]. The finding that hearts sourced from *Col19a1* null animals display de-repression of FAK signaling supports the notion that collagen XIX may inhibit postnatal cardiomyocyte growth by antagonizing myocyte integrin receptors and/or decreasing ECM/integrin receptor engagement.

Our study employed multiple design elements to promote rigor, demonstrate transparency, and enhance reproducibility. Mice were studied in a blinded manner using *a priori* application of ear tags, which were decoded only after the data were analyzed. No mice were excluded; all mice were reported herein; statistical design was rationalized before the experiments. Yet in terms of limitations, there were a few. To test the concept that FACITs can influence cardiac structure and function, we used a global *Col19a1* deficient mouse. There is no human condition related to global deficiency of *Col19a1*. Thus, we avoided making unreasonable translational claims. Most of our conclusions presumed fibroblasts were the sole/primary source of Col19a1 expression; however, given that smooth muscle cells also produce collagen XIX, it may also regulate vascular structure, and by extension, function. It is thus also possible that the developmental impact of constitutive *Col19a1* deficiency differed from (or was additive to) its role in the adult heart. Both possibilities will be addressed in future studies in which we use inducible, fibroblast-specific promoters.

In summary, we showed that collagen XIX impacts the structure and function of the heart. In addition, we showed that collagen XIX guides alignment, but not abundance, of fibrillar collagen. Such insights have important implications for cardiac development and the potential role of collagen XIX during acute, active tissue remodeling. In future studies, we will explore the role of collagen XIX during infarct-induced heart failure and test its role in reversible models of remodeling such as pregnancy-induced or exercise-elicited cardiac growth.

## Experimental procedures

All animal experiments were performed in accordance with the Guide for the Care and Use of Laboratory Animals published by the U.S. National Institutes of Health (Revised 2020) and were approved by the University of Louisville Institutional Animal Care and Use Committee (Louisville, KY, USA).

### Animals

Collagen XIX null mice (*Col19a1*^*N/N*^) on a C57BL/6 J background were kindly provided by Dr. Michael Fox (Fralin Biomedical Research Institute at Virginia Tech) and are previously described [50]. These mice harbor a *Col19a1* null mutation (denoted “N19”) that was generated by deleting exon 4 of collagen XIX [26]. All experiments in the study utilized collagen XIX nullizygous (*Col19a1*^*N/N*^) and wildtype littermate control (*Col19a1*^*+/+*^) mice at 18–20 weeks of age. Mice were housed in a pathogen-free facility under a standard 12 h light/dark cycle with ad libitum access to food and water. Genomic DNA was isolated from tails using an EZ Tissue/Tail PCR Genotyping Kit (EZ BioResearch LLC) according to the manufacturer instructions and genotyping was performed as previously described [50].

### Rigor, transparency, and reproducibility

A total of 20 mice were required for completion of *in vivo* experiments. This number was calculated via implementation of a statistical power analysis using SigmaStat Software (SigmaStat, Systat Software, Inc.). Based on preliminary experiments comparing cardiac function among *Col19a1* null and wildtype mice, we determined a 10.7% minimum detectable difference in means. Further, with these groups, a desired power of 80%, and an alpha of 0.05 (95th percentile), we calculated a requisite number of no fewer than 7 animals per group (*Col19a1*^*+/+*^ and *Col19a1*^*N/N*^). No animals were excluded from the study. All experiments were performed by a blinded observer—unapprised as to animal genotype/group assignment during outcome assessment and/or data analysis.

### Echocardiographic assessment of cardiac function

*Col19a1*^*N/N*^ and *Col19a1*^*+/+*^ littermates (both sexes) were subjected to echocardiography at 18–20 weeks of age. Transthoracic echocardiography of the left ventricle was performed as previously described [53]. The sonographer was blinded to mouse genotype at the time of assessment. Transthoracic echocardiography was performed using a Vevo 3100 echocardiography system. Mouse body temperature was maintained at 36.5–37.5 °C using a rectal thermometer interfaced with a servo-controlled heat lamp. Mice were anesthetized with 2% isoflurane and subsequently maintained under light anesthesia (≈1.5% isoflurane) during echocardiography procedures. Using the Vevo rail system, mice were placed in a supine position on an examination board interfaced with the Vevo 3100. M-mode, short-axis views of the left ventricle at the mid-papillary level allowed determination of left ventricular fractional shortening (FS), which was calculated by: $FS(\%)=\left(\frac{LVIDd-LVIDs}{LVIDd}\right)\times \;100$. B-mode, parasternal long-axis views of the left ventricle allowed determination of diastolic and systolic volumes. Stroke volume (SV) was calculated as: *SV*(*μl*) = *EDV − ESV*. Ejection Fraction (EF) was calculated as: $EF(\%)=\left(\frac{EDV-ESV}{EDV}\right)\times \;100$. Cardiac output (CO) was determined by: $CO\left(\frac{ml}{min}\right)=SV\times HR$.

### Cells, cell culture, and treatment

All cell lines were maintained under standard incubation conditions at 37 °C with 5% atmospheric CO_2_ and passaged using TrypLE™ (ThermoFisher Scientific) at 70–80% confluence.

### Cardiovascular cell isolation/procurement

Cardiac fibroblast isolation: cardiac fibroblasts were isolated from both male and female adult C57BL/6 J mice (18–20 wk) according to a previously established protocol with minor modification [23]. In brief, whole hearts were excised from isoflurane anesthetized mice, transferred to sterile 10 cm dishes, and mechanically minced using a razor blade. Minced myocardial tissues were transferred to a sterile 50 mL conical tube and washed via gravity sedimentation in 1 × PBS to remove red blood cells. Subsequently, tissues were enzymatically digested in 10 mL of filter sterilized (0.22 μm filter) collagenase II (1600 U/mL; Worthington Hills) at 37 °C for 25 min, with gentle agitation every 5 min. Enzymatic digestion was halted with the addition of 40 mL of mouse fibroblast complete medium [DMEM/F12, 10% FBS (VWR Life Science Seradigm Fetal Bovine Serum), 20 ng/mL bFGF (Peprotech), 20 ng/mL EGF (Peprotech), 1 × ITS (Thermo Fisher Scientific), 100 U/mL penicillin-streptomycin (Thermo Fisher Scientific), and 1 × GlutaMax (Thermo Fisher Scientific)]. Cells were then harvested by centrifugation at 600 × g for 10 min and then washed two times in complete medium. Lastly, cell pellets were gently resuspended in mouse fibroblast complete medium and grown in culture for 3–5 days prior to use.

Cardiomyocyte isolation: cardiomyocytes were isolated as previously [54] with minor modification. Briefly, adult C57BL/6 J mice (18–20 week) were intraperitoneally injected with 300 μL of 1000 U/mL heparin, placed aside for 10–15 min, and subsequently anesthetized by intraperitoneal administration of 120 μL of pentobarbital sodium (40 mg/mL). Hearts were immediately excised, rinsed with ice-cold 1 × PBS, aorta cannulated on a Langendorff apparatus, and secured via suture. Blood was cleared by perfusion with 1 × Tyrode’s solution (18 mM NaHCO_3_, 126 mM NaCl, 4.4 mM KCl, 1 mM MgCl_2_, 4 mM HEPES, 5.5 mM glucose, 10 mM 2,3-butanedione monoxime, 15 mM taurine, pH 7.4) and digestion carried out by addition of Liberase TH enzyme blend (0.28 mg/mL; Roche) for approximately 8 min. After perfusion, hearts were mechanically minced using fine forceps, followed by gentle trituration. Cell suspensions were filtered through 100 μm pore mesh to remove undigested tissue, and ventricular myocytes were allowed to settle by gravity (20 min). Cardiomyocytes were then sequentially resuspended and pelleted by centrifugation (500 × g for 4 min) in Tyrode’s solution (5 ×) containing increasing concentrations of Ca^2+^ (50 μM, 75 μM, 125 μM, 275 μM, and 525 μM). At the final step, cardiomyocytes were collected via brief centrifugation, suspended in plating medium (500 mL MEM, 1 μg/mL transferrin, 0.55 μg/mL selenium, 100 μg/mL penicillin-streptomycin, 2 mM glutamine, 4 mM NaHCO_3_, 10 mM HEPES, 0.2% BSA, 5% fetal bovine serum, and 10 mM 2,3-butanedione monoxime), and propagated in culture for 24 h prior to protein harvest.

Coronary smooth muscle cell isolation: aortas were removed from adult C57BL/6 J mice (18–20 wk) and placed in petri dishes containing 50 mL DMEM medium, supplemented with 20 μL Fungizone (Thermo Fisher Scientific; Cat. 15,290,018). Aortas were cut into 1 mm segments, transferred to 15 mL conical tubes, washed with Tyrode’s solution (7.37 mg/mL NaCl, 3.28 mg/mL KCl, 0.095 mg/mL MgCl_2_, 1.98 mg/mL glucose, 0.956 mg/mL HEPES, 1.51 mg/mL NaHCO_3_), and suspended in digestion buffer (1 mL Tyrode’s solution containing 2.2 mg of Collagenase type II and 1 μL of 20 mM CaCl_2_). Aortas were digested at 37 °C with periodic agitation for 30–60 min (until completely dissociated). Smooth muscle cells were harvested via brief centrifugation (300 × g for 5 min), suspended in plating medium (DMEM medium supplemented with 10% FBS, 1% penicillin-streptomycin), and propagated in culture for 2–3 days prior to protein harvest.

Coronary endothelial cells: C57BL/6 J primary coronary artery endothelial cells were sourced from Cell Biologics, Inc. (Cell biologics; Cat. C57–6093) and propagated in complete endothelial cell medium (Cell Biologics; Cat. 1168) according to the manufacturer’s instructions. Cells were grown in culture for 3–5 days prior to protein harvest.

### Immunoblotting

Immunoblotting was performed according to a previously described protocol [55] with minor modification of total protein isolation procedures. For the isolation of total protein extracts from cardiac tissue, flash-frozen myocardial specimens were suspended in RIPA lysis buffer (Thermo Fisher Scientific, Cat. 89,900) containing 1 × Halt™ Protease and Phosphatase Inhibitor Cocktail (Thermo Fisher Scientific, Cat. 78,444) at a ratio of 40 μL per mg of tissue. Tissue segments were then mechanically dissociated on ice using a handheld, electric tissue homogenizer followed with 10 s of sonication to facilitate dissociation of finer tissue aggregates. Samples were then incubated on ice for 30 min with periodic agitation and finally centrifuged at 14,000 × g for 15 min at 4 ° C to remove insoluble material. To prepare total pro-ein extracts from fibroblasts in culture, cells were trypsinized, washed twice with ice-cold 1 × PBS, and collected via centrifugation (500 × g for 5 min at 4 °C). Resulting cell pellets were suspended in 80–100 μL of RIPA lysis buffer containing protease and phosphatase inhibitors (as above) and mechanically lysed via repeated trituration on ice. Samples were then placed on ice for 30 min with intermittent agitation and then centrifuged at 14,000 × g for 15 min at 4 °C to remove insoluble cell debris. Resultant total protein lysates (from myocardial tissues and cells in culture) were analyzed for total protein content using a Pierce™ BCA Protein Assay Kit (Thermo Fisher Scientific; Cat. 23,225), according to the manufacturer’s instructions. Protein lysates were electrophoretically resolved on 4–12% Bis-Tris polyacrylamide gels (Thermo Fisher Scientific; Cat. NP0335), transferred to PVDF membranes (Thermo Fisher Scientific; Cat. LC2005), and blocked in 5% milk 1 × TBS-T overnight. Membranes were then rinsed with 1 × TBS-T, incubated with primary antibodies in antibody dilution buffer (5% BSA, 1 × TBS-T) at 4 °C, and lastly washed 5 times with 1 × TBS-T for 5 min each. Afterward, membranes were then incubated with HRP-conjugated secondary antibodies in 5% milk, 1 × TBS-T at room temperature and again washed 5 times with 1 × TBS-T for 5 min each. A detailed list of antibodies with corresponding dilutions is available in Supporting Information Table S1. HRP was detected using SuperSignal® West Pico Chemiluminescent Substrate (Thermo Fisher Scientific; Cat. 34,580) and digitally imaged using a Thermo Fisher Scientific myECL Imager. Images were subject to densitometric quantification using the ImageJ (version 1.53n) [56].

### Quantitative real-time PCR

Gene expression assays were performed as previously [55] with minor modification. Total RNA was isolated using a PureLink® RNA Mini Kit (Thermo Fisher Scientific) per the manufacturer’s instructions. 1000 ng of total RNA was reverse transcribed using the SuperScript™ IV VILO™ First-Strand Synthesis System (Thermo Fisher Scientific) containing both random hexamer and oligo(dT)18 primers, according to the manufacturer’s protocol. Quantitative PCR was performed with PerfeCTa® SYBR® Green FastMIX® in a QuantStudio^®^ 5 real-time PCR system (Thermo Fisher Scientific). Transcript specific primers and respective annealing temperatures are listed in Supporting Information Table S2. PCR was performed with an initial denaturation step of 95 °C for 10 min, followed by 40 cycles of 15 s denaturation at 95 °C and 1 min annealing/extension at 56–60 °C. Relative expression/fold expression was calculated according to the ΔΔC_T_ method for quantitative real-time PCR using *Actb* or *Gapdh* as an internal reference control where indicated.

### General histology and morphometry procedures

Hearts of isoflurane anesthetized mice were accessed by a thoracotomy and arrested in diastole via injection of 1 mL potassium chloride solution (3 M KCl in 1 × PBS) into the apex. Hearts were then removed and cannulated through the aorta using a Langendorff apparatus and retrogradely perfused with 1 × PBS for 3 min to clear blood within the coronary circulation, and perfused with 10% neutral buffered formalin solution for 10–15 min. Perfusion pressures were maintained between 60 and 80 mmHg. Following perfusion-fixation, total heart weight was measured on an analytical balance and tibia length determined using precision calipers. Heart weight (mg) to tibia length (mm) ratios were calculated and reported. Each heart was then cut into 3 transverse slices (each 3 mm thick), processed, paraffin-embedded, sectioned at 4 μm intervals, and subsequently utilized in histological and immunohistochemical analyses (detailed below).

### Determination of cardiomyocyte cross-sectional area

Mid-papillary level transverse myocardial sections were heated at 80 °C for 30 min, deparaffinized with xylene, and stepwise rehydrated by successive rinsing in decreasing concentrations of ethanol (100, 96, 90, and 80%) prior to placing in deionized water. Antigen retrieval was then performed by submerging tissue sections in citrate retrieval buffer [2.4 g/L sodium citrate tribasic dehydrate (S4641, Sigma-Aldrich), 0.35 g/L citric acid (C0759, Sigma-Aldrich), pH 6.0] for 10 min at approximately 100 °C. Sections were allowed to cool for 10 min on ice and washed in deionized water prior to application of diluted antibodies and wheat germ agglutinin (WGA) (detailed in Supporting Information Table S3). Slides were incubated with α-sarcomeric actin primary antibody in antibody diluent reagent (Thermo Fisher Scientific, Cat. 003,218) for 1 h at 37 °C and then washed three times with 1 × PBS. Sections were then simultaneously incubated with Alexa Fluor 633 conjugated secondary antibody and Alexa Fluor 555 conjugated WGA in antibody diluent reagent for 1 h at 37 °C. After three washes with 1 × PBS, slides were counterstained with 1 μg/mL DAPI (D3571, Thermo Fisher Scientific) for 10 min and subsequently incubated with 0.1% Sudan Black solution (Sigma-Aldrich, Cat. 199,664) in 70% ethanol for 15 min to mitigate autofluorescence signal. Sections were then washed with 1 × PBS (three times), rinsed in deionized water, and finally mounted under glass coverslips using PermaFluor Aqueous Mounting Medium (Thermo Fisher Scientific, Cat. TA-030-FM). Entire myocardial sections were imaged, without regional bias, using a Keyence BZ-X810 microscope with 49,000-UF1 C195675 (DAPI), 49,008_UF1 C195528 (Texas Red), 49,009-UF1 C195677 (Cy5) filter cubes. Cardiomyocyte cross-sectional areas were calculated in an automated fashion using the Keyence BZ-X800 Analyzer Software with the following conditions: nucleated cardiomyocytes (DAPI^+^), circularity ≥0.50, and area range of 50–600 nm.

### Nidogen 1 basement membrane staining

As above, transverse myocardial tissue sections heated at 80 °C, deparaffinized with xylene, and stepwise rehydrated. Samples were then permeabilized with 0.2% Triton X-100 in 1 × PBS for 15 min, rinsed with deionized water for 3 min, and subjected to citrate buffer retrieval procedures (as detailed above). Sections were cooled on ice for 10 min, rinsed in deionized water, and blocked with 2% donkey serum (Immuno Reagents Inc., Cat. SP-072-VX10) for 2 h at room temperature. Subsequently, slides were washed once with 1 × PBS and then incubated with diluted nidogen 1 antibody in diluent reagent (Thermo Fisher Scientific, Cat. 003,218) overnight at 4 °C. The following day, slides were washed with 1 × PBS (three times) and incubated with Alexa Fluor 488 conjugated secondary antibody in antibody diluent reagent for 1 h at 37 °C. After three washes with 1 × PBS, slides were incubated with α-sarcomeric actin antibody for 1 h at 37 °C, washed three times with 1 × PBS, and finally incubated with Alexa fluor 633 secondary antibody in antibody diluent reagent for 1 h at 37 °C. Sections were then counterstained with 1 μg/mL DAPI (D3571, Thermo Fisher Scientific) for 10 min and incubated with 0.1% Sudan black solution (Sigma-Aldrich, Cat. 199,664) in 70% ethanol for 15 min to reduce autofluorescence signal. Sections were washed with 1 × PBS (three times), rinsed in deionized water, and mounted under glass coverslips using PermaFluor Aqueous Mounting Medium (ThermoFisher Scientific, Cat. TA-030-FM). Whole myocardial tissue sections were imaged using a Keyence BZ-X810 microscope with 49,000-UF1 C195675 (DAPI), 49,002-UF1 C195676(GFP), 49,009-UF1 C195677 (Cy5) filter cubes. All antibody information and dilutions used are detailed in Supporting Information Table S3.

### Sirius red/fast green collagen staining

Mid-papillary level transverse myocardial sections were deparaffinized and rehydrated as above. Sirius red/fast green stain was prepared using 0.1% (w/v) Direct Red 80 (Cat. 365,548–5G, Sigma-Aldrich) and 0.1% Fast Green FCF (w/v) (Cat. F7252, Sigma-Aldrich) in picric acid (P6744-1GA, Sigma-Aldrich). Myocardial sections were incubated in sirius red/fast green solution for 30 min at room temperature, rinsed in deionized water, dehydrated with 100% ethanol, rinsed in xylene, and mounted under glass coverslips using Permount Mounting Media (Cat.17986-05, Electron Microscopy Sciences). Brightfield images were digitally acquired using a Keyence BZ-X810 microscope using both 20 × and 4 × objectives.

### Neonatal mouse cardiomyocyte proliferation and myocardial apoptosis

Isoflurane anesthetized mouse pups at postnatal day 3 were decapitated, their hearts quickly removed, and placed in 10% neutral buffered formalin solution overnight. The following day neonatal hearts were processed via an automated tissue processor, paraffin-embedded, and sectioned at 4 μm intervals. Coronal plane cardiac tissue sections were deparaffinized, rehydrated, permeabilized, and subjected to antigen retrieval procedures (as described above). Slides were then blocked with 2% donkey serum (Immuno Reagents Inc., Cat. SP-072-VX10) for 2 h at room temperature and rinsed with 1 × PBS prior to application of diluted antibodies (detailed in Supporting Information Table S3). For the assessment of cardiomyocyte proliferation, slides were incubated with anti-phospho-histone H3 antibody, diluted in antibody diluent reagent (Thermo Fisher Scientific, Cat. 003,218), overnight at 4 °C. Sections were then washed three times with 1 × PBS, incubated with Alexa Fluor 488 secondary antibody in diluent reagent for 1 h at 37 °C, and again washed three times with 1 × PBS. Slides were subsequently incubated with anti-α-sarcomeric actin antibody for 1 h at 37 °C, washed three times with 1 × PBS, and incubated with Alexa Fluor 633 secondary antibody in antibody diluent reagent for 1 h at 37 °C. After three washes with 1 × PBS, slides were counterstained with 1 μg/mL DAPI (D3571, Thermo Fisher Scientific) for 10 min and then incubated with 0.1% Sudan Black solution (Sigma-Aldrich, Cat. 199,664) in 70% ethanol for 15 min. Sections were then washed with 1 × PBS (three times), rinsed in deionized water, and mounted under glass coverslips using PermaFluor Aqueous Mounting Medium (Thermo Fisher Scientific, Cat. TA-030-FM). Entire myocardial sections were imaged, without regional bias, using a Keyence BZ-X810 microscope with 49,000-UF1 C195675 (DAPI), 49,002_UF1 C195676 (GFP), 49,009-UF1 C195677 (Cy5) filter cubes. For the evaluation of myocardial apoptosis, coronal tissue sections were subjected to TUNEL (terminal deoxynucleotidyl transferase dUTP nick-end labeling). Tissue sections were stained using the Click-iT™ TUNEL Assay for *In Situ* Apoptosis Detection kit, Alexa Fluor 488 dye (Thermo Fisher Scientific, Cat. C10617), with strict adherence to the manufacturer’s protocol. Resultant sections were imaged, without regional bias, using a Keyence BZ-X810 microscope with 49,000-UF1 C195675 (DAPI) and 49,002_UF1 C195676 (GFP) filter cubes. The number of pH3^+^ or TUNEL^+^ cells were enumerated in ImageJ (version 1.53n) [56] by a blinded observer unaware of genotype.

### Myocardial hydroxyproline content

Myocardial collagen content was enumerated using a colorimetric hydroxyproline assay kit (Abcam, Cat. Ab222941) according to the manufacturer’s instructions. Briefly, 100 mg of isolated heart tissue was homogenized in 74 μl of distilled water using a 1 mL Dounce tissue grinder (Sigma-Aldrich, Cat. DWK885300). Next, 100 μL of 10 N NaOH was added to homogenates, heated for 1 h at 120 °C, and neutralized via the addition of 100 μL of 10 N HCl. Samples were then centrifuged at 10,000 × g to remove insoluble material. Resultant supernatants were placed into 96 well plates (10 μL per well) and crystallized by heating at 65 °C. Subsequently, 100 μL of oxidation solution (consisting of 96 μL oxidation buffer and 4 μL chloramine T concentrate) was added to each well and incubated at room temperature for 20 min. Then 50 μL of developer solution was added to each well and placed at 37 °C for 5 min. Afterward, 50 μL of 4-(dimethylamino)benzaldehyde (DMAB) solution was pipetted per well and incubated at 65 °C for 45 min. Sample absorbance values were measured at 560 nm on a SynergyMx plate reader (BioTek) and compared to kit provided standards to calculate total myocardial hydroxyproline content in μg per mg of wet tissue.

### *In vitro* collagen matrix organization

Approximately 300,000 cardiac fibroblasts were plated in 10 cm tissue culture grade dishes and grown for ten days in fibroblast complete medium. Cells were then counterstained via the addition of Alexa Fluor 555 conjugated WGA diluted in 1 × PBS (1 μL/mL). After a 5 min incubation, plates were rinsed with 1 × PBS, and subjected to second harmonic generation (SHG) imaging using a Nikon A1R MP+ multiphoton microscope with an Apo LWD 25 × immersion objective. The excitation laser was tuned to 950 nm and SHG signal was collected through a FITC bandpass filter with an emission wavelength of 525 nm. Square images were acquired with 1024 × 1024 pixels of resolution with a scan speed of approximately 15 s. Resultant SHG images were subjected to computational analyses using MATLAB software framework packages, CurveAlign and CT-FIRE [57].

### Transmission electron microscopy of decellularized hearts

Hearts of isoflurane anesthetized mice were accessed by a thoracotomy. Hearts were then removed, aortas positioned onto a cannula attached to a peristaltic pump (Grainger, Cat. 20LX53), and affixed via a suture loop. Hearts were retrogradely perfused with 1 × PBS for 10 min followed by decellularization solution A (1% SDS, 1 × PBS) for 24 h. The following day, hearts were perfused with decellularization solution B (0.07% Triton X-100, 1% SDS, 1 × PBS) for an additional 24 h and finally rinsed with 1 × PBS for 15 min. Decellularized hearts were then removed, minced into 1 mm^3^ cubes using a razor blade, and incubated with tissue fixative (2% glutaraldehyde, 2% paraformaldehyde, 0.1 M monosodium phosphate in deionized water; pH 7.4) at 4 °C for 72 h. Samples were then suspended in osmium tetroxide solution (1% osmium tetroxide, 0.1 M monosodium phosphate in deionized water; pH 7.4) at room temperature for 2 h and subsequently washed with 0.1 M monosodium phosphate. Decellularized tissues were then stepwise dehydrated by successive incubation in increasing concentrations of ethanol (50, 70, 95, and 100%). Following dehydration, samples were infiltrated with a 1:1 and then 1:3 mixture of absolute ethanol and resin [5.7 g Durcupan component A (Sigma-Aldrich, Cat. 44,611), 5.5 g Durcupan component B (Sigma-Aldrich, Cat. 44,612), 0.34 g Durcupan component C (Sigma-Aldrich, Cat. 44,613), and 0.16 g Durcupan component D (Sigma-Aldrich, Cat. 44,610/44,614)] for 1 h each. Following the removal of the 1:3 alcohol/resin mix, pure resin was added and samples placed in a compressor chamber overnight. Resin capsules were polymerized in a 60 °C oven for 2 d. Embedded samples were sectioned to a thickness of 70 nm on a Leica EM UC7 ultramicrotome and collected on nickel grids. Grids were then stained with 1% uranyl acetate for 15 min, rinsed, and stained with Reynolds’ lead citrate. Grids were then rinsed and dried. Images were acquired using a Hitachi HT7700 Transmission Electron Microscope at 80 kV.

### Statistical analyses

All statistical analyses were performed using GraphPad Prism version 7.05 for Windows (GraphPad Software, La Jolla California USA, http://www.graphpad.com). Data distribution characteristics were evaluated using the Shapiro–Wilk test for normality. Data sets following a Gaussian distribution were evaluated using an unpaired, two-tailed Student’s T-test. Non-normally distributed data were analyzed via a non-parametric Mann–Whitney U test. In all cases, *p*-values of less than 0.05 were considered statistically significant.

## Supplementary Material

1

2

## Figures and Tables

**Table 1.** Indices of cardiac structure and function in *Col19a1* wildtype (+/+) and null (N/N) mice

	+/+ (*N* = 13)	N/N (*N* = 11)	*p*-value
Sex (female:male)	7:6	9:2	
HR (bpm)	529 ± 9	519 ± 10	0.3607
SV (μL)	23.1 ± 1.3	23.2 ± 0.9	0.9173
CO (mL/min)	12.2 ± 0.7	12.1 ± 0.6	0.8928
LVAWd (mm)	1.1 ± 0.1	0.9 ± 0.0	*0.0394
LVAWs (mm)	1.6 ± 0.1	1.4 ± 0.1	*0.0136
LVPWd (mm)	1.0 ± 0.0	0.9 ± 0.0	*0.0101
LVPWs (mm)	1.7 ± 0.1	1.4 ± 0.1	*0.0002
LVIDd (mm)	3.0 ± 0.1	3.2 ± 0.1	0.3228
LVIDs (mm)	1.7 ± 0.1	2.0 ± 0.1	*0.0156
FS (%)	45 ± 1	37 ± 2	*0.0057

* Echocardiographic measures of cardiac structure and function in *Col19a1* wildtype (+/+) and null (N/N) mice at 18–20 wk of age. Endpoints assessed include: heart rate (HR), stroke volume (SV), cardiac outputt (CO), left ventricular anterior (LVAW) and posterior wall thickness (LVPW) in diastole (d) and systole (s), left ventricular internal diameter (LVID) in diastole (d) and systole (s), and fractional shortening (FS). Tables depict arithmetic mean ± SEM. Statistics: Data normality was assessed using the Shapiro–Wilk test. SV, CO, LVPAWs, LVPWd, LVPWs, LVIDd, LVIDs, and FS were analyzed by an unpaired, two-tailed Student’s T-test. HR and LVAWd were analyzed using the non-parametric Mann–Whitney U test. *p* < 0.05 was considered statistically significant and are highlighted by an asterisks (*).

## Abbreviations:

CF: cardiac fibroblast
CM: cardiomyocyte
CO: cardiac output
EC: endothelial cell
EDV: end diastolic volume
EF: ejection fraction
ESV: end systolic volume
FS: fractional shortening
HR: heart rate
HW: heart weight
LVAW: left ventricular anterior wall
LVID: left ventricular internal diameter
LVPW: left ventricular posterior wall
SM: smooth muscle
SV: stroke volume
TL: tibia length
WGA: wheat germ agglutinin
