# Supplementary material for: Collagen type XIX regulates cardiac extracellular matrix structure and ventricular function
Source: Matrix Biol. Author manuscript; Available in PMC 2022 Jun 2. (PMC9161575; doi:10.1016/j.matbio.2022.03.007)
Supplement: 1 [file NIHMS1808927-supplement-1.docx]

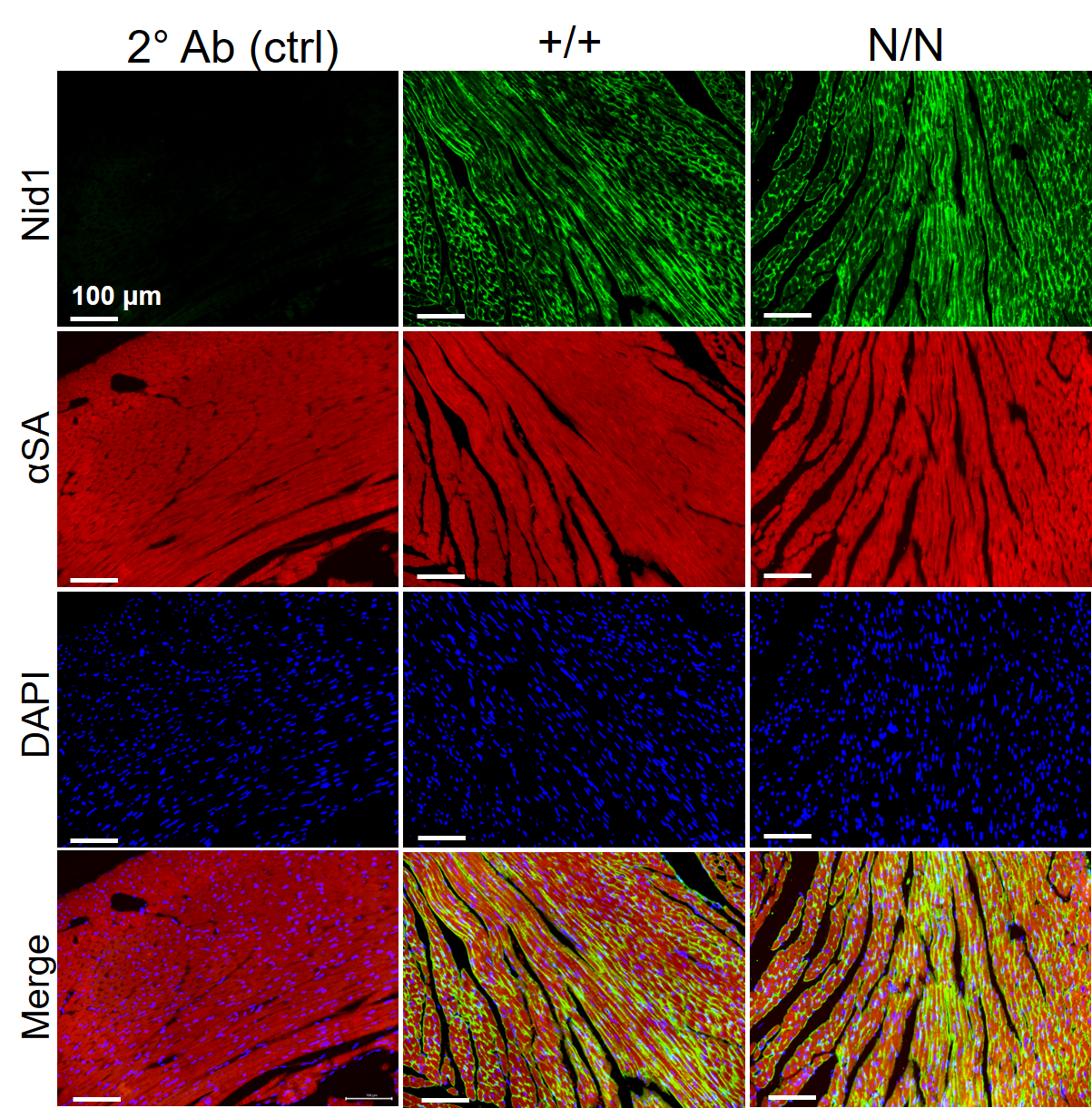


**Supplementary Figure S1: Basement membrane morphology is preserved in myocardium of *Col19a1* null animals.** Images depict transverse myocardial tissue sections sourced from *Col19a1* wildtype (+/+, N=4 [2 female, 2 male]) and null (N/N; N=4 [2 female, 2 male]) mice. Sections were stained with antibodies against nidogen 1 (Nid1; green) and α-sarcomeric actin (αSA; myocytes; red). Nuclei were counterstained with DAPI (nuclei; blue).


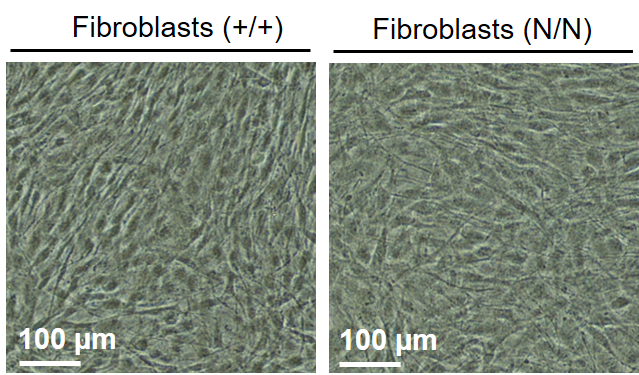


**Supplementary Figure S2: *Col19a1* null and wildtype cardiac fibroblast morphology in culture.** Phase contrast images of cardiac fibroblasts isolated from *Col19a1* wildtype (+/+; left panel) and null (N/N; right panel) mice at day 7 in culture prior to second-harmonic generation imaging.


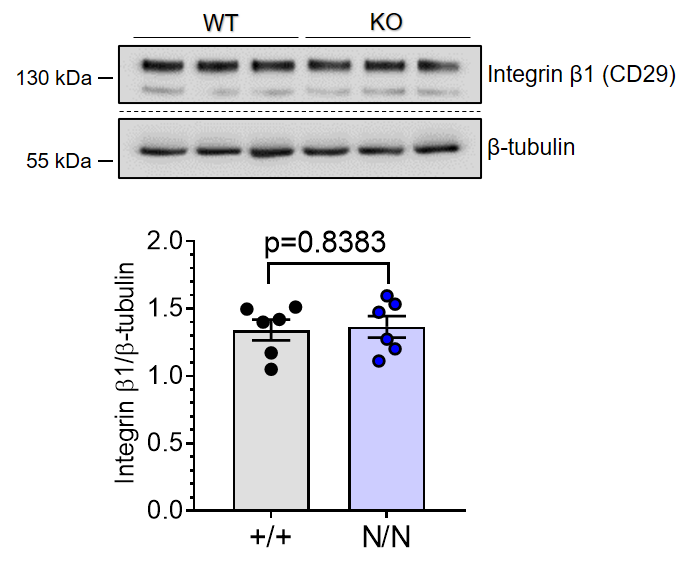


**Supplementary Figure S3: Cardiac Integrin β1 expression is unaltered in *Col19a1* nullizygous mice.** Representative Westerns evaluate the expression of integrin β1 in myocardial tissues from *Col19a1* wildtype (+/+) and null (N/N) mice at 18–20 wk. β-tubulin is a loading control. Immunoblot markers denote molecular weight standards (in kDa) resolved with experimental protein lysates. Graphs report total integrin β1 levels relative to β-tubulin ± SEM. N=6 independent biological replicates per group [3 female, 3 male]. Statistics: Data normality was assessed using the Shapiro–Wilk test. Data were analyzed by an unpaired, two-tailed Student’s T-test. p<0.05 was considered statistically significant.
